# Pore-Throat-Adaptive Transport and Flow-Redistribution Mechanisms of Deformable Hydrogel Particles in Tight Reservoirs of the Ordos Basin

**DOI:** 10.3390/gels12100924

**Published:** 2026-10-14

**Authors:** Hongguang Li, Hujun Gong

**Affiliations:** 1 Qingyang Exploration and Development Branch, Liaohe Oilfield Company, Yan’an 716200, China; xltcyc_yangyz@petrochina.com.cn; 2 State Key Laboratory of Continental Evolution and Early Life, Northwest University, Xi’an 710069, China

**Keywords:** deformable hydrogel particles, tight reservoirs, pore-throat matching, flow redistribution, movable fluid redistribution

## Abstract

Tight reservoirs commonly exhibit strong pore-throat confinement and flow heterogeneity, which cause injected water to preferentially migrate through connected high-conductivity pathways while a large fraction of movable fluids remains under-swept. To address this limitation, deformable hydrogel particles (DHPs) with tunable particle size and network density were prepared through inverse emulsion polymerization using acrylamide, 2-acrylamido-2-methylpropane sulfonic acid, acrylic acid, and N,N′-methylenebisacrylamide. The chemical structure, porous morphology, swelling behavior, deformation recovery, pore-throat matching, core-scale transport, and flow-redistribution performance were systematically investigated using Fourier transform infrared spectroscopy (FTIR), scanning electron microscopy (SEM), laser particle size analysis, compression testing, mercury intrusion porosimetry, nuclear magnetic resonance (NMR), micro-computed tomography (micro-CT), and core flooding. The results show that emulsification speed regulated the hydrated particle size, while crosslinking density controlled the internal network compactness and deformation capacity. The hydrated D50 values of the small-, medium-, and large-sized DHPs (DHP-S, DHP-M, and DHP-L) were 58.3 ± 3.5, 118.6 ± 6.9, and 224.7 ± 12.5 μm, respectively. Among the tested samples, DHP-M showed a balanced swelling ratio of 4.63 ± 0.31, a compressive stress of 14.8 ± 1.1 kPa at 50% strain, and an elastic recovery ratio of 81.6 ± 3.2%. A bulk strain-adjusted apparent confinement analysis identified DHP-M as providing a more suitable balance between pore entry and selective retention; however, the apparent confinement coefficient was used only as a comparative descriptor and not as a literal single-particle geometric pass/fail criterion. Core-flooding experiments further showed that DHP-M formed distributed retention in intermediate pore-throat systems and produced a residual resistance factor of 8.2. In heterogeneous parallel core systems, DHP-M preferentially retained in high-flow branches, redirecting chase water toward low-flow branches. Matched brine-only controls produced less than 2.5 percentage points of additional oil recovery, whereas DHP-M treatment increased the original oil in place (OOIP)-based recovery factor by 11.8–16.4 percentage points. This work provides a laboratory-scale pore-throat-adaptive design strategy for deformable hydrogel particles and demonstrates their potential for selective flow redistribution and sweep-efficiency improvement in tight heterogeneous reservoirs.

## 1. Introduction

Tight sandstone reservoirs have become an important target for unconventional oil development, but their efficient exploitation remains limited by strong pore-throat confinement and pronounced flow heterogeneity. In the Ordos Basin, tight reservoirs commonly contain micron- to submicron-scale throats, complex pore-throat connectivity, and a large fraction of bound or poorly movable fluids. Recent studies combining mercury intrusion porosimetry, nuclear magnetic resonance (NMR), micro-computed tomography, and microscopic imaging have shown that pore-throat architecture controls both reservoir seepage capacity and movable fluid occurrence [1,2,3,4,5,6,7,8]. In such reservoirs, permeability is not governed only by total porosity, but depends more strongly on throat size, connected-pore accessibility, and the spatial continuity of preferential flow pathways [2,4,6]. Consequently, conventional water flooding tends to advance through relatively connected high-conductivity channels, while tighter pore-throat domains remain weakly swept. This mismatch between injected-water accessibility and movable fluid distribution is one of the main reasons for limited sweep efficiency in tight heterogeneous reservoirs.

Conformance-control materials, especially polyacrylamide gels, preformed particle gels (PPGs), microgels, and related hydrogel particles, have been widely investigated as chemical agents for reducing excessive water channeling and improving sweep efficiency in heterogeneous reservoirs [9,10,11,12,13,14,15]. Compared with in situ gel systems, preformed hydrogel particles can be synthesized, size-controlled, and evaluated before injection, which provides better controllability over swelling, strength, and plugging behavior [9,10,16,17]. Recent studies have developed various polymer-based and biomass-derived PPG systems, including carboxymethyl cellulose-based gels, microwave-assisted polyacrylamide gels, nanomaterial-reinforced particle gels, agar-based particle gels, and hydrophobically modified acrylamide hydrogel particles [18,19,20,21,22,23,24]. These materials can increase flow resistance in high-permeability pathways and redirect injected water toward less-swept regions. Visualized flooding experiments have further shown that PPGs can migrate, pile up, deform, and re-migrate in porous media, indicating that particle transport is a dynamic process rather than a simple static plugging event [14]. Related pore-scale visualization studies in hydrogel-modified porous media have also shown that local flow pathways and particle retention can be strongly influenced by hydrogel-induced network entrapment and pore-scale structural barriers [25].

Despite these advances, applying hydrogel particles to tight reservoirs remains challenging because the characteristic throat size is usually much smaller than the hydrated particle size. If hydrogel particles are too small or too weak, they may pass through connected channels without forming effective retention. If they are too large or too rigid, they may accumulate near the inlet and cause shallow plugging, which limits deep flow regulation. Therefore, particle size design alone is insufficient for tight reservoirs. The deformability, elastic recovery, swelling stability, and pore-throat matching relationship of hydrogel particles must be considered together. Recent research on nanometer plugging agents, gel sealing materials, degradable liquid plugs, interfacial reinforcement of gel systems, and shale-well plugging/inhibition materials has emphasized the importance of matching material structure with pore-scale or interface-scale confinement [26,27,28,29,30]. However, a quantitative framework linking hydrated particle size, deformation-assisted size reduction, throat diameter, retention location, and movable fluid redistribution remains insufficiently established for tight sandstone systems.

The acrylamide (AM)/2-acrylamido-2-methylpropane sulfonic acid (AMPS)/acrylic acid (AA) hydrogel network provides a suitable platform for designing deformable particles for saline reservoir environments. Acrylamide contributes a flexible polymer backbone, 2-acrylamido-2-methylpropane sulfonic acid introduces strongly hydrophilic and salt-tolerant sulfonic groups, and acrylic acid provides ionizable carboxyl groups for swelling regulation. By adjusting emulsification conditions and crosslinking density, hydrogel particles with different hydrated sizes, internal network compactness, and deformation recovery can be obtained. However, for tight reservoirs, the key scientific issue is not only whether such particles can swell or plug, but whether they can enter accessible connected pathways, deform at local throat constrictions, recover sufficiently to form retention, and finally redirect subsequent water toward under-swept movable fluid domains.

In this study, deformable AM/AMPS/AA hydrogel particles were prepared by inverse emulsion polymerization and evaluated for pore-throat-adaptive transport and deep flow diversion in Ordos tight cores. The chemical structure, internal network morphology, particle size evolution, swelling behavior, and deformation recovery of the hydrogel particles were first characterized. The pore-throat architecture and movable fluid distribution of representative tight sandstone cores were then analyzed using mercury intrusion porosimetry, NMR, and micro-CT. A deformation-corrected pore-throat matching coefficient was introduced to evaluate the apparent matching relationship between hydrated particles and tight core throats. Finally, single-core transport tests, heterogeneous parallel core flow diversion experiments, and movable fluid redistribution measurements were conducted to clarify the relationship among deformable migration, selective retention, flow redistribution, and displacement enhancement. This work aims to provide a material design and matching evaluation strategy for hydrogel-assisted deep flow diversion in tight heterogeneous reservoirs.

## 2. Results and Discussion

### 2.1. Structural Verification and Particle Size Regulation of Deformable Hydrogel Particles

#### 2.1.1. Chemical Structure Analysis

**Figure 1:**
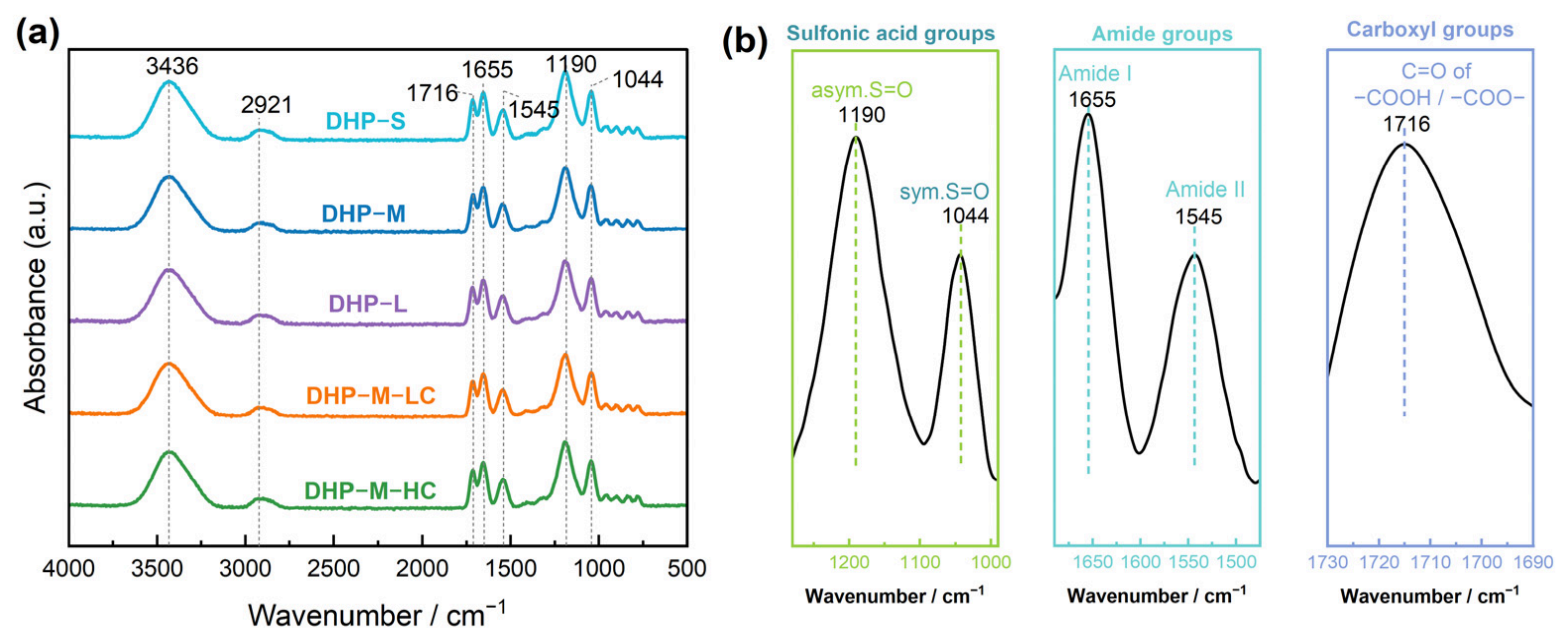
FTIR characterization of deformable hydrogel particles: (**a**) full FTIR spectra of DHP-S, DHP-M, DHP-L, DHP-M-LC, and DHP-M-HC; (**b**) enlarged characteristic absorption regions for amide, sulfonic acid, and carboxyl groups. The colors in panel (**b**) indicate functional-group categories rather than sample identities.

Fourier transform infrared spectroscopy (FTIR) analysis was used to identify the principal functional groups of the deformable hydrogel particles and to evaluate whether their spectra were consistent with the expected AM/AMPS/AA-based network structure. As shown in Figure 1a, DHP-S, DHP-M, DHP-L, DHP-M-LC, and DHP-M-HC exhibited similar absorption profiles, indicating similar chemical functionalities among the prepared hydrogel formulations. The broad band at 3430–3442 cm^−1^ was assigned to –NH/–OH stretching vibrations, which originated mainly from amide groups in AM and AMPS and carboxyl-containing units from AA. These polar groups provided the primary hydration sites of the hydrogel network.

The bands at 1652–1658 cm^−1^ and 1542–1548 cm^−1^ corresponded to Amide I and Amide II vibrations, respectively, confirming the presence of acrylamide-based units in the crosslinked network. The peaks at 1186–1195 cm^−1^ and 1038–1048 cm^−1^ were attributed to asymmetric and symmetric S=O stretching vibrations in sulfonic acid groups, indicating the incorporation of AMPS units. In addition, the absorption band at 1710–1722 cm^−1^ was assigned to C=O stretching of –COOH/–COO^−^ groups from AA. These characteristic bands support the presence of amide, sulfonic acid, and carboxyl functionalities in the hydrogel particles.

Figure 1b further magnifies the characteristic absorption regions of amide, sulfonic acid, and carboxyl groups, allowing the overlapped bands in the 1800–1000 cm^−1^ region to be distinguished more clearly. No obvious residual vinyl C=C absorption was observed, suggesting that the vinyl groups of AM, AMPS, AA, and MBAA participated in free-radical polymerization. However, the absence of a detectable vinyl C=C band in the FTIR spectra should not be interpreted as quantitative evidence of complete monomer conversion, because low levels of residual unsaturation may remain below the detection sensitivity of the measurement. Meanwhile, DHP-M-LC, DHP-M, and DHP-M-HC showed no new absorption bands, indicating that changing MBAA dosage regulated the network density without altering the primary chemical structure.

These FTIR results are consistent with the formation of AM/AMPS/AA-based hydrogel particles containing the expected hydrophilic and ionic functionalities. The principal FTIR absorption bands and their corresponding assignments are summarized in Table 1. These identified functional groups provide the chemical basis for the observed hydration and salt-tolerant behavior, while the influence of network morphology and particle size is examined below.

#### 2.1.2. Porous Morphology and Crosslinking-Density-Regulated Network Architecture

**Figure 2:**
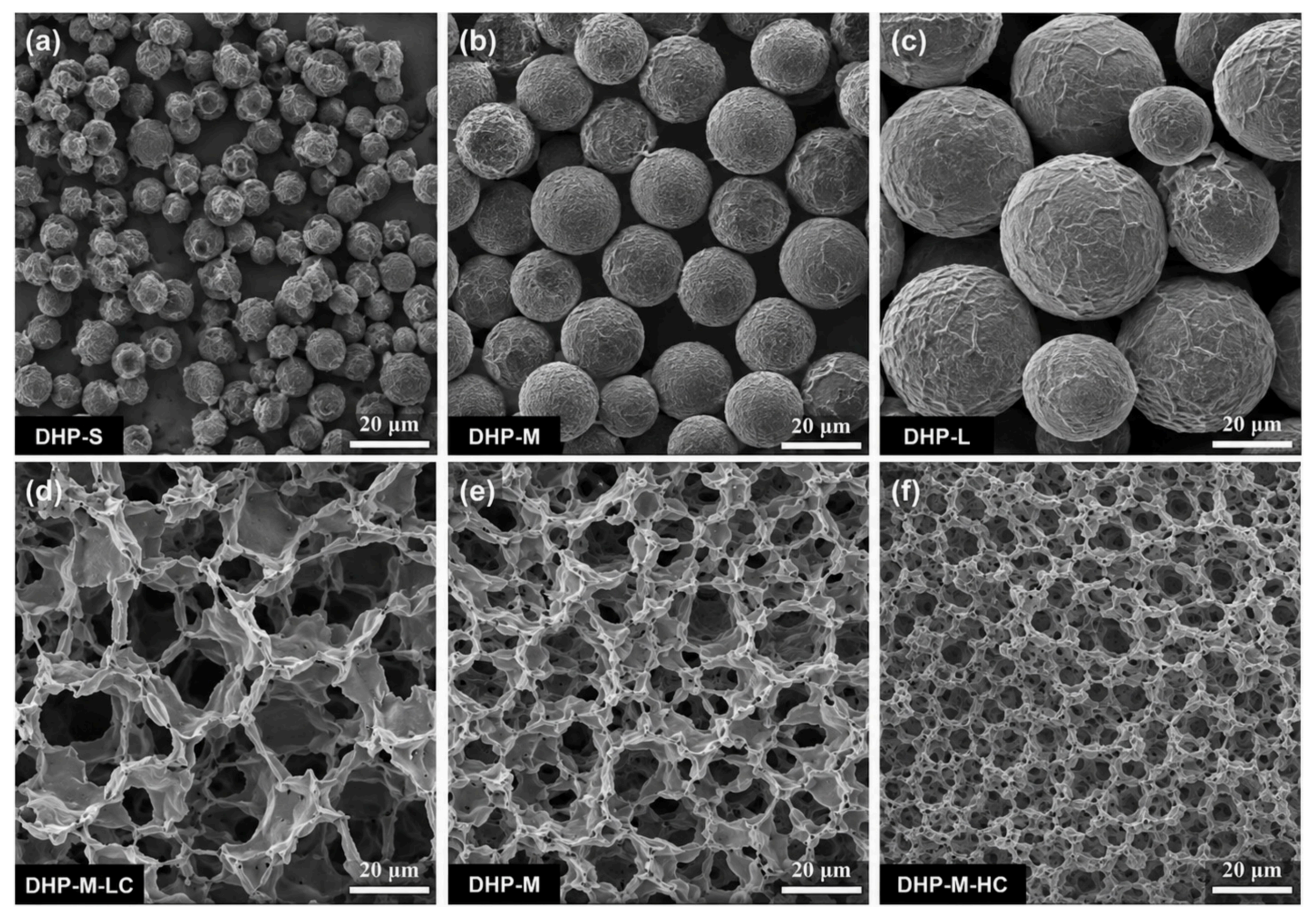
SEM morphology of deformable hydrogel particles: (**a**–**c**) overall particle morphology of DHP-S, DHP-M, and DHP-L prepared under different emulsification speeds at the same MBAA dosage of 0.30 wt%; (**d**–**f**) freeze-dried internal network morphology of DHP-M-LC, DHP-M, and DHP-M-HC prepared with different MBAA dosages. Pore-size data are expressed as mean ± standard deviation from three independent synthesis batches (*n* = 3); multiple SEM fields were averaged within each batch.

Scanning electron microscopy (SEM) was used to examine the particle morphology and internal network structure. As shown in Figure 2a–c, DHP-S, DHP-M, and DHP-L retained discrete particle morphologies rather than forming continuous bulk gels. The particles were generally spherical or irregularly spherical, with rough surfaces and local shrinkage after drying. This morphology is consistent with inverse emulsion polymerization and is suitable for preparing injectable particle suspensions.

At the same MBAA dosage of 0.30 wt% of the total monomer mass, the particle size changed with emulsification speed. DHP-S mainly consisted of smaller particles, DHP-M showed an intermediate size, and DHP-L contained larger particles with more evident surface wrinkling. This result indicates that emulsification speed controlled the initial droplet size and consequently regulated the final hydrogel particle size.

The internal porous structure was further compared using DHP-M-LC, DHP-M, and DHP-M-HC. As shown in Figure 2d–f, increasing the MBAA dosage made the network more compact after freeze-drying. DHP-M-LC, prepared with 0.20 wt% MBAA, showed a loose network with an average pore diameter of 18.7 ± 2.3 μm. DHP-M, prepared with 0.30 wt% MBAA, exhibited a moderately porous structure with an average pore diameter of 12.9 ± 1.6 μm. When the MBAA dosage increased to 0.50 wt%, DHP-M-HC formed a denser skeleton, and the average pore diameter decreased to 7.6 ± 1.1 μm. The corresponding SEM-derived pore-structure parameters are summarized in Table 2.

The decrease in the SEM-derived pore diameter is consistent with increasing crosslinking density. A lower MBAA dosage allowed greater network expansion during hydration, whereas a higher dosage restricted chain extension and produced a compact skeleton after freeze-drying. Because freezing and subsequent freeze-drying can alter the dimensions and arrangement of a hydrated polymer network, the pore diameters measured from Figure 2d–f should be interpreted as comparative descriptors of the freeze-dried microstructure rather than as direct measurements of pore dimensions in the fully hydrated state. Nevertheless, all samples were subjected to the same freezing, freeze-drying, and SEM preparation procedure; therefore, the systematic decrease in apparent pore size from DHP-M-LC to DHP-M-HC remains useful for comparing relative network compactness among the three crosslinking levels. Hydrated-state behavior was evaluated independently through swelling, hydrated particle size, compression, and elastic-recovery measurements in the subsequent sections. For pore-throat transport, this network difference is important because a loose network favors compression and shape adjustment, while a dense network provides higher rigidity but lower deformation capacity. Therefore, the swelling and recovery behavior of these particles was further evaluated in Section 2.2.

#### 2.1.3. Particle Size Regulation Through Inverse Emulsion Control

**Figure 3:**
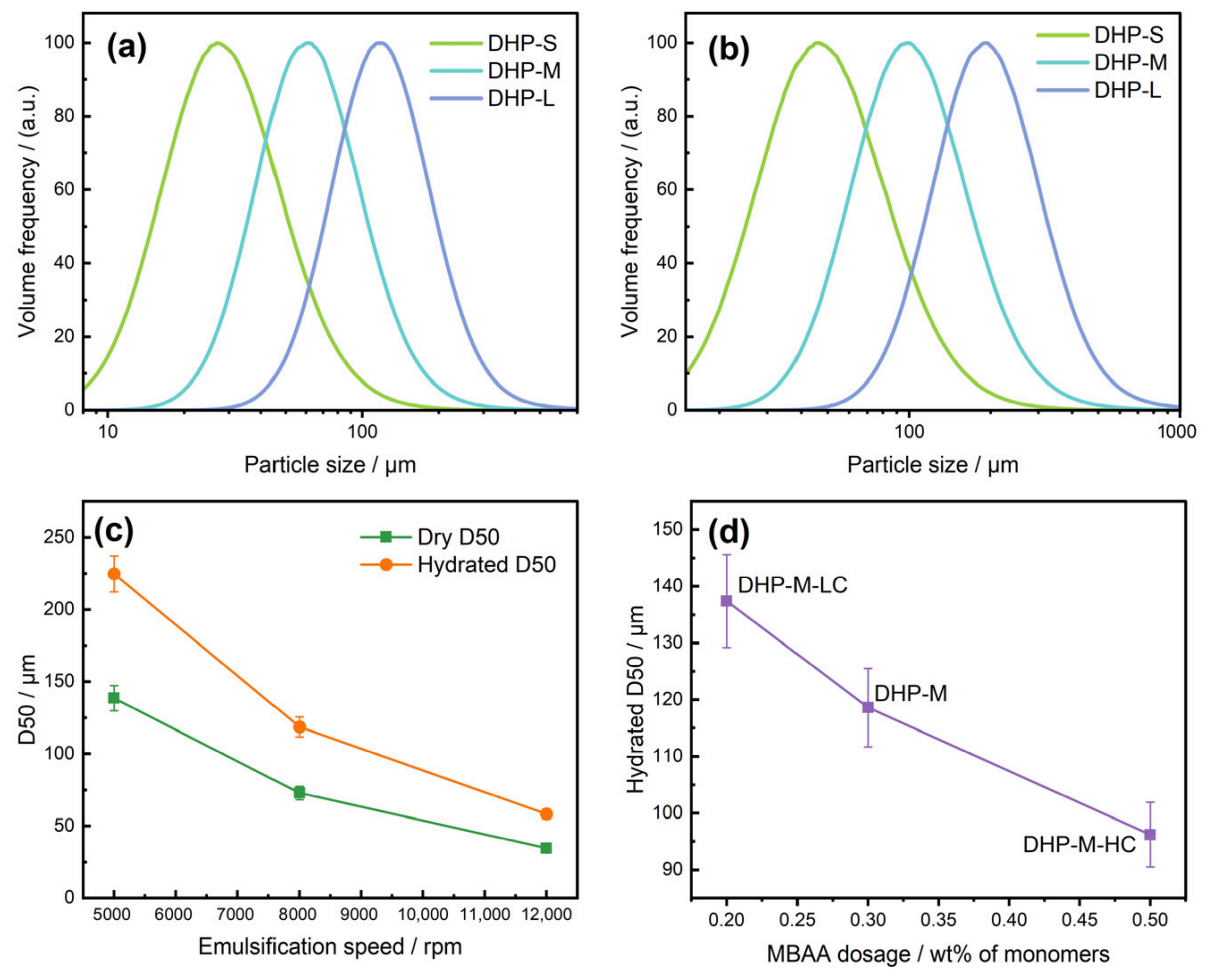
Particle size regulation of deformable hydrogel particles measured by laser particle size analysis: (**a**) dry particle size distributions of DHP-S, DHP-M, and DHP-L prepared at different emulsification speeds; (**b**) hydrated particle size distributions after swelling in simulated formation water at 60 °C for 24 h; (**c**) dry and hydrated D50 values as a function of emulsification speed; (**d**) effect of MBAA dosage on hydrated D50 of medium-sized hydrogel particles. Data are expressed as mean ± standard deviation from three independent synthesis batches (*n* = 3).

Particle size is a key parameter controlling the entry, migration, and retention of hydrogel particles in tight pore-throat systems. As shown in Figure 3a, the dry particle size distributions of DHP-S, DHP-M, and DHP-L shifted toward larger sizes as the emulsification speed decreased from 12,000 to 5000 rpm. The dry D50 values were 34.6 ± 2.1, 72.8 ± 4.6, and 138.5 ± 8.7 μm, respectively, confirming that emulsification speed effectively regulated the initial particle size level. The complete dry and hydrated particle size parameters of the five hydrogel formulations are summarized in Table 3.

After hydration in simulated formation water at 60 °C for 24 h, the particle size distributions shifted to larger ranges (Figure 3b). The hydrated D50 values of DHP-S, DHP-M, and DHP-L increased to 58.3 ± 3.5, 118.6 ± 6.9, and 224.7 ± 12.5 μm, respectively. The size order remained unchanged after swelling, indicating that the particle size hierarchy formed during emulsification was preserved during hydration. Because the particles were injected into tight cores in a hydrated state, the hydrated particle size was used for subsequent pore-throat matching analysis.

The hydrated Span values of DHP-S, DHP-M, and DHP-L were 1.33, 1.19, and 1.11, respectively. These values indicate that the particles were not narrowly monodisperse, but the distribution widths remained controllable. D50 was therefore used as the representative particle size, while D10 and D90 were retained to describe the influence of distribution breadth.

Figure 3c shows that both dry and hydrated D50 decreased with increasing emulsification speed, consistent with droplet breakup in inverse emulsion systems. Higher shear intensity promoted droplet fragmentation and produced smaller particles. Hydration increased the D50 of all samples, with a larger absolute increase for larger particles. For example, DHP-L increased from 138.5 μm in the dry state to 224.7 μm after hydration, whereas DHP-S increased from 34.6 to 58.3 μm.

The effect of crosslinking density was examined using DHP-M-LC, DHP-M, and DHP-M-HC. Their dry D50 values were close, ranging from 71.6 ± 4.2 to 74.1 ± 4.8 μm, indicating that MBAA dosage had limited influence on initial droplet size under the same emulsification condition. After hydration, however, DHP-M-LC showed the largest hydrated D50 of 137.4 ± 8.2 μm, DHP-M-HC decreased to 96.2 ± 5.7 μm, and DHP-M remained intermediate at 118.6 ± 6.9 μm. This result agrees with the SEM-observed network structures: lower crosslinking density promoted swelling-induced expansion, whereas higher crosslinking density restricted particle growth.

The controlled-series design distinguishes the roles of emulsification speed and crosslinking density more clearly. Within the constant-crosslinking series (0.30 wt% MBAA), increasing the emulsification speed from 5000 to 12,000 rpm progressively reduced the dry D50 from 138.5 to 34.6 μm, and the corresponding particle size hierarchy was retained after hydration. Thus, emulsification speed acted as the upstream processing variable governing particle size formation, whereas particle size represents the resulting structural state rather than an experimentally independent synthesis factor. In contrast, at a fixed emulsification speed of 8000 rpm, changing the MBAA dosage produced only a limited variation in dry D50 but markedly altered the hydrated D50, indicating that crosslinking density primarily regulated swelling-induced network expansion after particle formation.

The subsequent core-flooding comparison therefore evaluates the transport consequences of the three emulsification-speed-generated particle size classes under identical pore-throat and injection conditions, while the DHP-M-LC/DHP-M/DHP-M-HC series is used specifically to evaluate crosslinking-controlled swelling and mechanical behavior.

### 2.2. Swelling Behavior and Deformation Recovery Under Simulated Reservoir Conditions

Swelling and deformation recovery determine whether hydrogel particles can maintain an appropriate hydrated size and adapt to pore-throat confinement during injection. Particles with insufficient swelling may not form effective retention, whereas excessive swelling or poor recovery may increase the risk of near-inlet accumulation. Therefore, the swelling kinetics, short-term temperature and salinity responses, and compression recovery of the prepared particles were evaluated under simulated reservoir conditions.

**Figure 4:**
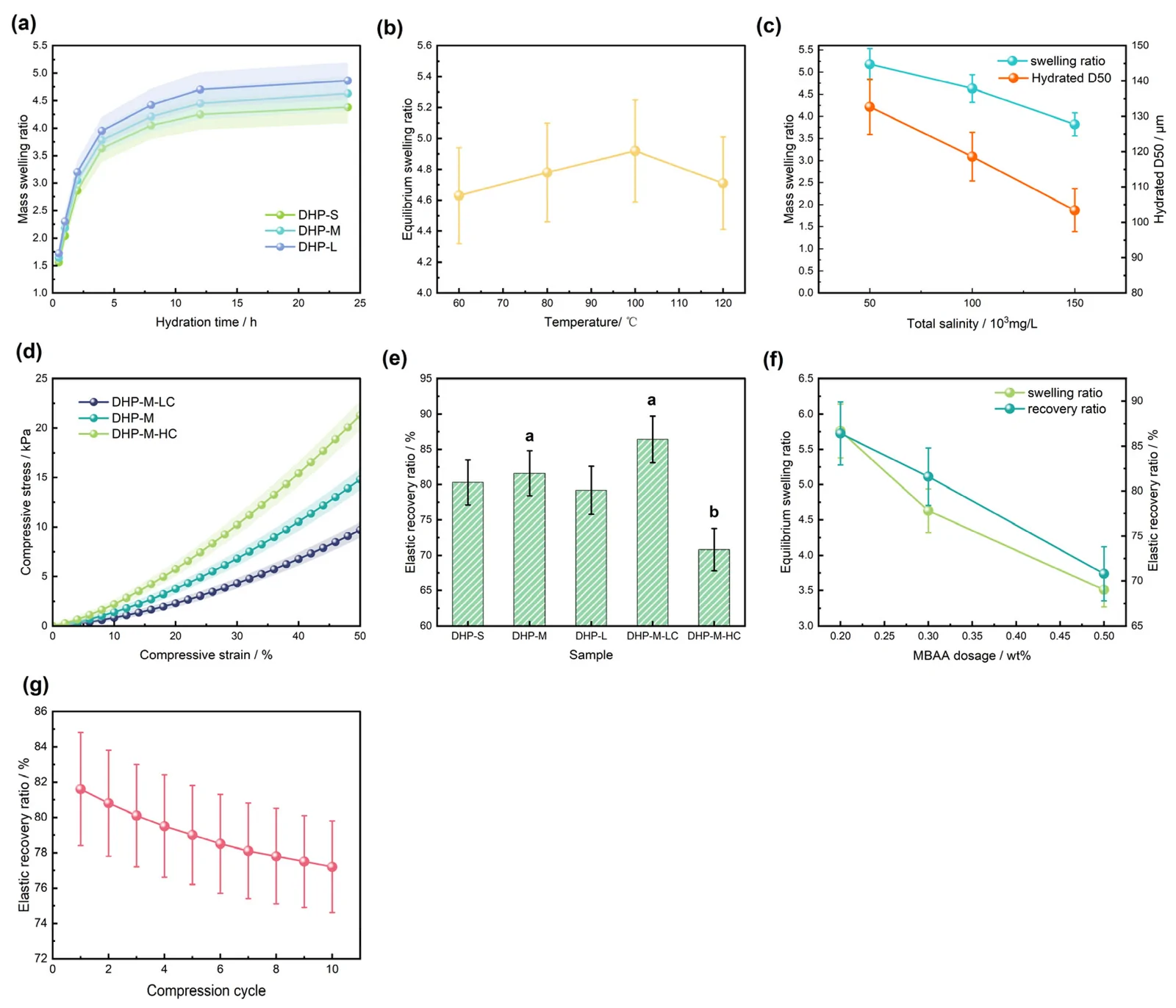
Swelling and deformation recovery behavior of deformable hydrogel particles under simulated reservoir conditions: (**a**) swelling kinetics of DHP-S, DHP-M, and DHP-L at 60 °C; (**b**) effect of temperature on the equilibrium swelling ratio of DHP-M after 24 h; (**c**) effect of salinity on the swelling ratio and hydrated D50 of DHP-M; (**d**) compression stress–strain curves of DHP-M-LC, DHP-M, and DHP-M-HC; (**e**) elastic recovery ratios after 50% compression; (**f**) comparison of swelling ratio and recovery ratio for medium-sized particles with different MBAA dosages. (**g**) cyclic elastic recovery of DHP-M during ten consecutive compression–unloading cycles at 50% maximum strain. Data are expressed as mean ± standard deviation from three independent synthesis batches (*n* = 3). Different lowercase letters in panel (**e**) indicate significant differences according to Tukey’s HSD test (*p* < 0.05).

As shown in Figure 4a, DHP-S, DHP-M, and DHP-L all showed a rapid swelling stage during the initial hydration period, followed by a slower approach to equilibrium. For DHP-M, the swelling ratio increased from 2.18 ± 0.16 at 1 h to 4.21 ± 0.27 at 8 h and reached 4.63 ± 0.31 after 24 h. DHP-S and DHP-L exhibited similar swelling profiles, with equilibrium swelling ratios of 4.38 ± 0.29 and 4.86 ± 0.34, respectively. The limited difference among the three size fractions indicates that emulsification speed mainly regulated particle size, while the basic swelling capacity was controlled by the same AM/AMPS/AA network.

Temperature had a moderate effect on the equilibrium swelling ratio of DHP-M. As shown in Figure 4b, the swelling ratio increased from 4.47 ± 0.28 at 60 °C to 4.92 ± 0.33 at 100 °C and then slightly decreased to 4.71 ± 0.30 at 120 °C. This variation suggests that elevated temperature promoted chain mobility and water diffusion within a limited range, while excessive temperature may partially restrict network expansion. Over the 24 h test period, DHP-M maintained comparable hydration levels across the tested temperature range, indicating limited short-term sensitivity to temperature under these conditions.

The salinity effect was more pronounced than the temperature effect. As shown in Figure 4c, increasing the total salinity from 50,000 to 150,000 mg/L reduced the swelling ratio of DHP-M from 5.18 ± 0.35 to 3.82 ± 0.26, while the hydrated D50 decreased from 132.7 ± 7.8 to 103.4 ± 6.1 μm. The decrease can be attributed to reduced osmotic pressure difference and ionic shielding of charged groups in the polymer network. A similar salinity-dependent suppression of hydrogel swelling has been reported for reservoir-oriented preformed particle gels, where increasing ionic strength reduced water uptake by screening charged polymer groups and decreasing the osmotic driving force for network expansion [13,20]. However, the magnitude of the salinity response depends strongly on polymer composition, crosslinking density, and reinforcing components. The present DHP-M retained a hydrated D50 above 100 μm even at 150,000 mg/L, indicating that the AMPS-containing network preserved substantial short-term hydration capacity under the tested high-salinity conditions. Nevertheless, DHP-M still maintained measurable swelling and a hydrated D50 above 100 μm at 150,000 mg/L, showing that the AMPS-containing network retained substantial hydration capacity after 24 h exposure to high-salinity water. Because the temperature- and salinity-response tests were limited to 24 h, these results describe short-term hydration behavior and should not be interpreted as evidence of long-term reservoir stability. Extended aging under coupled high-temperature and high-salinity conditions is required to evaluate long-term structural and swelling stability. Consistent with the salinity-dependent change in hydrated particle size, the 0.30 wt% DHP-M dispersion maintained low bulk viscosity over the tested salinity range (Table S6). At 100,000 mg/L and 60 °C, the apparent viscosity decreased from 3.46 mPa·s at 10 s^−1^ to 2.52 mPa·s at 100 s^−1^, indicating only mild shear-thinning behavior and confirming that the observed core-scale pressure response was governed primarily by particle–pore interactions rather than by high bulk-fluid viscosity.

The influence of crosslinking density was further evaluated using DHP-M-LC, DHP-M, and DHP-M-HC. As summarized in Table 4, DHP-M-LC showed the highest equilibrium swelling ratio of 5.76 ± 0.38, whereas DHP-M-HC showed the lowest value of 3.51 ± 0.24. DHP-M remained intermediate at 4.63 ± 0.31. This trend is consistent with the SEM results: a lower MBAA dosage produced a looser network and stronger swelling, while a higher crosslinking density restricted chain extension and water uptake.

Compression tests were then used to evaluate particle deformation behavior. As shown in Figure 4d, the hydrated particle beds exhibited nonlinear stress–strain responses, with stress increasing more rapidly at higher strain. At 50% strain, the compressive stress increased from 9.7 ± 0.8 kPa for DHP-M-LC to 14.8 ± 1.1 kPa for DHP-M and 21.3 ± 1.5 kPa for DHP-M-HC. This increase reflects the enhanced rigidity caused by higher crosslinking density.

The elastic recovery results in Figure 4e show the opposite trend. DHP-M-LC exhibited the highest recovery ratio of 86.4 ± 3.5%, while DHP-M-HC decreased to 70.8 ± 3.0%. DHP-M achieved an intermediate recovery ratio of 81.6 ± 3.2%. The effect of crosslinking density on elastic recovery was statistically significant (one-way ANOVA, F(2, 6) = 18.246, *p* = 0.002815). Tukey’s HSD test showed that DHP-M-HC differed significantly from DHP-M-LC (*p* = 0.002556) and DHP-M (*p* = 0.015273), whereas the difference between DHP-M-LC and DHP-M was not significant (*p* = 0.243354). Although DHP-M-LC recovered more readily after compression, its high swelling and low compression resistance may increase the risk of particle accumulation in confined throats. DHP-M-HC provided stronger mechanical support but had lower recoverability, which may limit adaptive deformation during transport.

Figure 4f compares the swelling ratio and elastic recovery ratio of the medium-sized particles with different crosslinking densities. DHP-M did not show the highest swelling ratio or the highest rigidity, but it provided a balanced combination of hydration capacity, compression resistance, and deformation recovery. This balance supports its selection as the representative hydrogel particle for subsequent pore-throat matching and core-scale transport experiments.

To further evaluate whether DHP-M retained recoverable deformation under repeated mechanical loading, ten consecutive compression–unloading cycles were performed at a maximum strain of 50%. As shown in Figure 4g, the elastic recovery ratio decreased only moderately from 81.6% in the first cycle to 77.2% in the tenth cycle. Meanwhile, the compressive stress at 50% strain decreased from 14.8 to 13.2 kPa, retaining approximately 89% of the initial load-bearing response. After an additional 10 min of unloaded recovery following the tenth cycle, the recovery ratio increased to 79.6%, indicating that part of the cycle-induced deformation remained time-dependent and reversible. The limited attenuation in both recovery and compressive response suggests progressive mechanical accommodation rather than rapid structural fatigue, demonstrating that DHP-M maintained substantial recoverable deformability during repeated loading.

Because cyclic compression of a hydrated particle bed does not reproduce the local geometry, particle–wall contact, friction, and fluid redistribution associated with individual pore-throat extrusion, the result is interpreted as a mechanical measure of repeated-deformation tolerance rather than as a direct simulation of repeated pore-throat passage.

### 2.3. Pore-Throat Architecture and Movable Fluid Distribution of Ordos Tight Cores

The pore-throat architecture of tight cores determines both the accessibility of hydrogel particles and the distribution of movable fluids. To provide the structural basis for subsequent pore-throat matching and transport analysis, Ordos tight sandstone (OTS) cores were classified into OTS-L, OTS-M, and OTS-H groups, representing progressively increased pore-throat confinement and reduced flow accessibility. The complete physical and mineralogical properties of the individual core plugs used throughout the experiments are provided in Supplementary Table S1, whereas Table 5 summarizes the group-level pore-throat characteristics used for comparison.

**Figure 5:**
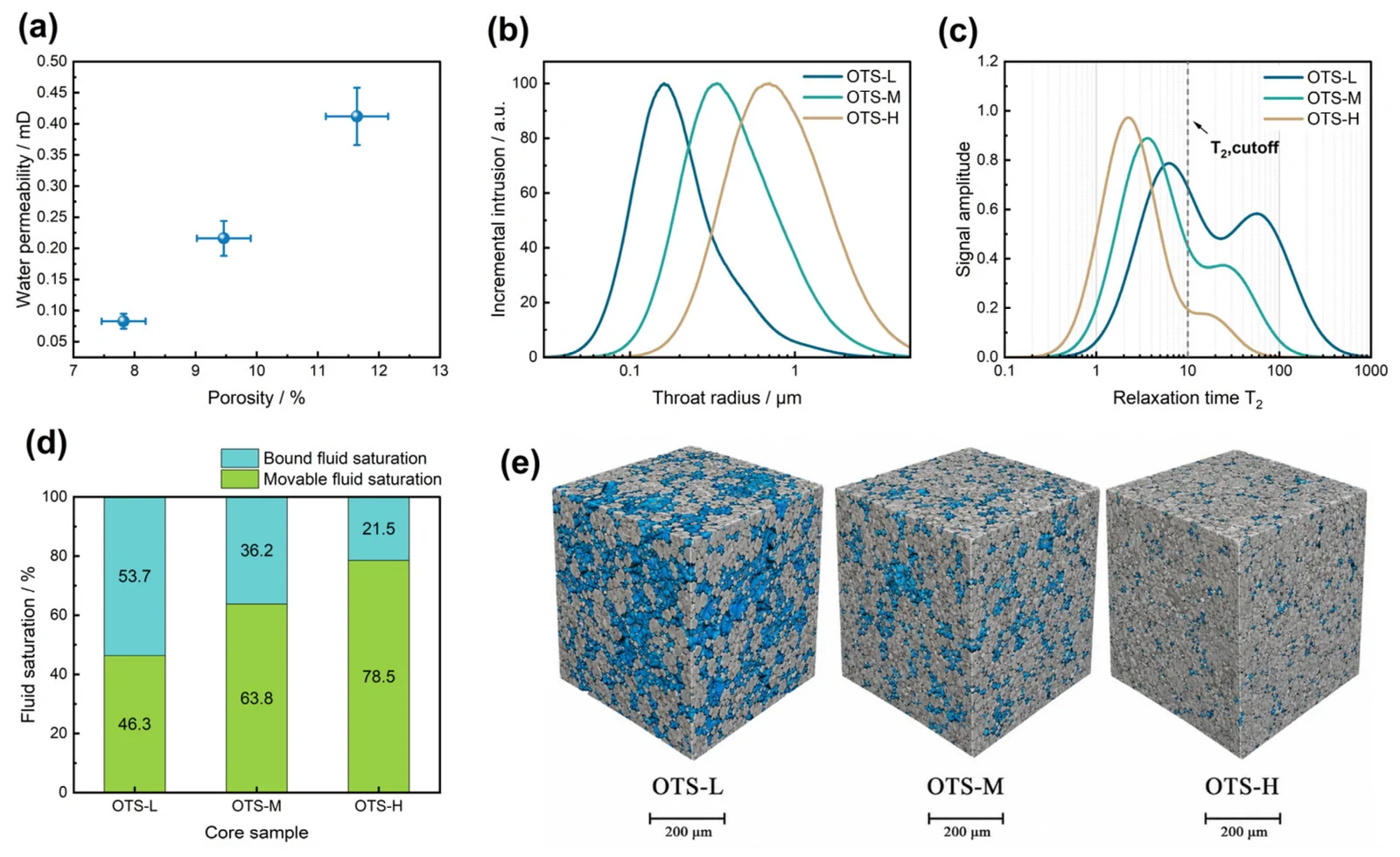
Pore-throat architecture and movable fluid distribution of Ordos tight cores: (**a**) porosity–permeability relationship of OTS-L, OTS-M, and OTS-H cores; (**b**) throat-radius distributions obtained from mercury intrusion porosimetry; (**c**) NMR T_2_ spectra of fully saturated cores; (**d**) bound and movable fluid saturations derived from NMR measurements; (**e**) representative micro-CT-based 3D reconstructions of resolvable connected pore pathways in the three core groups (voxel size: 8.0 μm). In panel (**e**), the gray phase represents the rock matrix, while the blue phase represents segmented connected pore space. Quantitative data are expressed as mean ± standard deviation from three independent core plugs per group (*n* = 3); panel (**e**) shows representative micro-CT reconstructions.

As shown in Figure 5a, OTS-L showed the most favorable storage–flow capacity, whereas OTS-H exhibited the strongest restriction to fluid transport. The porosity decreased from 11.64 ± 0.51% in OTS-L to 9.46 ± 0.44% in OTS-M and 7.82 ± 0.36% in OTS-H. Meanwhile, the water permeability decreased more sharply from 0.412 ± 0.046 mD to 0.216 ± 0.028 mD and 0.083 ± 0.012 mD, respectively. The stronger decrease in permeability than in porosity indicates that flow capacity was controlled not only by pore volume but also by throat connectivity and local constriction.

Mercury intrusion porosimetry further confirmed the throat-scale differences among the three core groups. As shown in Figure 5b, the median throat radius decreased from 0.62 ± 0.07 μm in OTS-L to 0.34 ± 0.04 μm in OTS-M and 0.18 ± 0.03 μm in OTS-H. The corresponding characteristic throat diameters were 1.24 ± 0.14, 0.68 ± 0.08, and 0.36 ± 0.06 μm, respectively. This narrowing of throat size from OTS-L to OTS-H indicates a gradual reduction in the accessible migration window for hydrated hydrogel particles.

The NMR T_2_ spectra in Figure 5c provided additional information on the relative degree of fluid confinement within the pore system. OTS-L exhibited a broader T_2_ distribution and a more evident long-relaxation component, indicating a greater proportion of fluid residing in relatively weakly confined and hydraulically accessible pore domains. In contrast, OTS-H was dominated by short-T_2_ signals, indicating stronger fluid–surface interaction and a greater proportion of fluid in highly confined pore domains. OTS-M showed an intermediate distribution, consistent with its transitional pore-throat structure. Because surface relaxivity was not independently calibrated, these T_2_ components were interpreted comparatively and were not assigned directly to absolute pore-size classes.

The NMR-derived fluid saturation results in Figure 5d further quantified this difference. The movable fluid saturation decreased from 53.7 ± 2.6% in OTS-L to 36.2 ± 2.9% in OTS-M and 21.5 ± 3.1% in OTS-H, while the bound fluid saturation increased from 46.3 ± 2.6% to 63.8 ± 2.9% and 78.5 ± 3.1%, respectively. These results show that the tighter cores did not simply have lower pore volume; rather, a larger fraction of their pore space became hydraulically ineffective or poorly movable.

The micro-CT reconstructions in Figure 5e visually support the above interpretation at the resolvable micro-CT scale. OTS-L contained more abundant and continuous connected pore spaces, which agrees with its higher permeability and movable fluid saturation. OTS-M showed a transitional pore network, where connected pathways and local throat constrictions coexisted. OTS-H presented sparse and discontinuous connected pores, indicating limited deep migration space for hydrogel particles and a higher probability of localized retention. This interpretation is consistent with previous multiscale NMR and CT observations of hydrogel-modified sand, where hydrogel incorporation altered pore occupancy, pore connectivity, and the spatial distribution of hydrogel-filled regions within the porous structure [31].

Importantly, these observations indicate that the mercury intrusion porosimetry (MIP)-derived median throat diameter should not be regarded as the unique transport-controlling aperture. Hydrogel-particle migration is governed by the accessible connected flow backbone rather than by an isolated statistical throat size. Relatively large and hydraulically connected pores or channels provide the principal migration space, whereas local critical constrictions connecting these larger pore bodies act as bottlenecks that control particle deformation, transient bridging, retention, and subsequent re-migration. Hydraulically connected microfractures, if present, may provide additional preferential pathways with effective apertures substantially larger than the median matrix-throat diameter. However, because the present MIP and micro-CT measurements do not resolve or quantify every sub-resolution constriction and microfracture, their contribution is considered qualitatively rather than assigned a separate quantitative matching parameter.

Taken together, the transition from OTS-L to OTS-H reflects a progressive reduction in pore-throat connectivity, characteristic throat diameter, and movable fluid availability. These structural differences provide the reservoir basis for evaluating hydrogel–pore-throat matching. In relatively connected cores, hydrogel particles may migrate more deeply before retention, whereas in highly confined cores, narrow throats may promote shallow retention or inlet-biased accumulation.

### 2.4. Pore-Throat Matching Between Deformable Hydrogels and Ordos Tight Cores

Pore-throat matching was evaluated to clarify whether the hydrated hydrogel particles could enter, deform, and become retained in tight core pore systems. Because hydrogel particles are deformable rather than rigid grains, both the hydrated particle size and compression-induced size reduction were considered in the matching analysis. The key hydrogel and core parameters used for the pore-throat matching analysis are summarized in Table 6.

**Figure 6:**
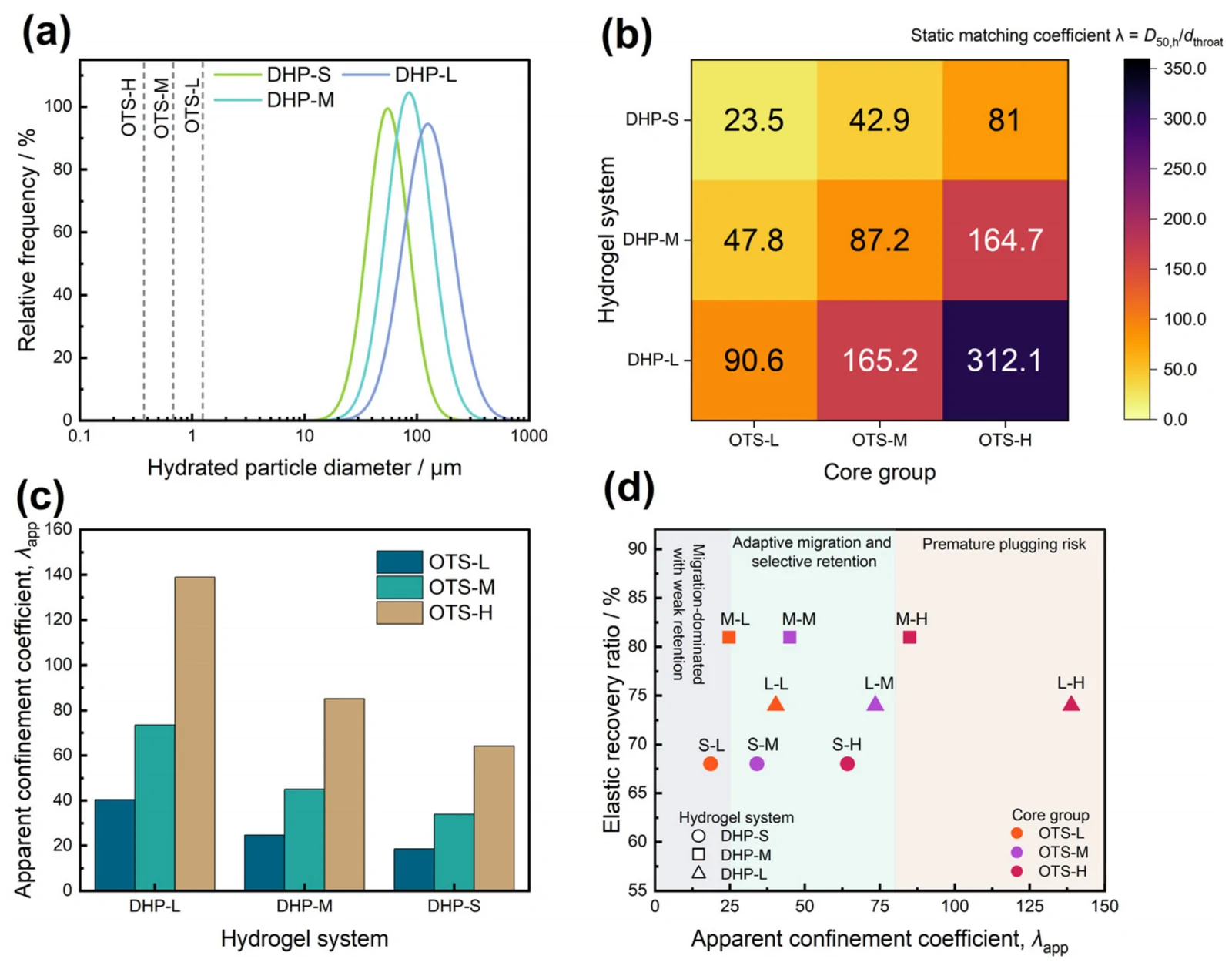
Pore-throat matching relationship between deformable hydrogels and Ordos tight cores: (**a**) hydrated particle size distributions of DHP-S, DHP-M, and DHP-L compared with the characteristic throat diameters of OTS-L, OTS-M, and OTS-H; (**b**) particle/throat size matching coefficients for different hydrogel–core combinations; (**c**) bulk strain-adjusted apparent confinement coefficients; (**d**) matching-regime map showing easy passage with weak retention, adaptive migration with selective retention, and premature plugging risk. In panel (**d**), *λ*_app_ = 25 and 80 represent empirical, dataset-specific operational boundaries separating the three transport regimes and are not intended as universal critical matching coefficients.

As shown in Figure 6a, the hydrated *D*50 values of DHP-S, DHP-M, and DHP-L were 58.3 ± 3.5, 118.6 ± 6.9, and 224.7 ± 12.5 μm, respectively. By contrast, the characteristic throat diameters of OTS-L, OTS-M, and OTS-H were only 1.24 ± 0.14, 0.68 ± 0.08, and 0.36 ± 0.06 μm, respectively. This pronounced dimensional mismatch indicates that the characteristic throat diameter cannot be interpreted as a literal geometric passage aperture for individual hydrogel particles. In the present analysis, *d*_throat_ is defined as twice the median throat radius derived from mercury intrusion porosimetry and therefore represents the central tendency of the throat-size population rather than the aperture of every hydraulically connected pathway. In this framework, *d*_throat_ is retained only as a scalar reference for comparing relative confinement among the tested hydrogel–core combinations. Neither *λ* nor *λ*_app_ is intended to represent the aperture of the actual controlling transport pathway, which depends on the connectivity of larger accessible pores, the dimensions of local critical constrictions along the percolating pathway, and any hydraulically connected fracture-like features that may be present. These network-scale factors were therefore interpreted jointly with the MIP throat-size distributions and micro-CT reconstructions rather than replaced by the median throat diameter alone. Accordingly, the experimentally observed particle transport does not imply that intact hydrated particles freely traverse 0.36–1.24 μm throats. Instead, transport is considered to be selective and to occur primarily through the relatively larger and better-connected subset of the pore-throat network, whereas finer local constrictions promote transient deformation, bridging, retention, and pressure-driven re-migration. Such dynamic transport modes are consistent with the deform-and-pass, snap-off-and-pass, and trap behaviors previously reported for preformed particle gels in porous media [10,12].

The static particle/throat matching coefficient, λ, was calculated as *D*50/*d*_throat_. As shown in Figure 6b, *λ* increased as the core became tighter. For DHP-S, *λ* increased from 47.0 in OTS-L to 85.7 in OTS-M and 161.9 in OTS-H. For DHP-M, the corresponding values were 95.6, 174.4, and 329.4, while DHP-L showed higher values of 181.2, 330.4, and 624.2. These results show that the static size mismatch became more severe from OTS-L to OTS-H. However, static λ values tend to overestimate the restriction to transport because they do not include particle deformability.

To further account for deformation, a standardized bulk strain normalization was applied to provide a comparative description of the relative confinement of the three hydrogel size fractions. Using the 50% bulk-compression reference state, the apparent size proxies, *D*_app_, of DHP-S, DHP-M, and DHP-L were 29.2, 59.3, and 112.4 μm, respectively, and the corresponding apparent confinement coefficient was defined as *λ*_app_ = *D*_app_/*d*_throat_. These values do not represent the actual diameters attained by individual hydrogel particles inside pore throats. Rather, they describe how the initial particle size mismatch would scale under an identical macroscopic compression reference condition. It should be emphasized that the 50% strain was obtained from compression of a hydrated particle bed rather than from direct measurement of single-particle deformation during extrusion through a calibrated constriction. The bed strain includes not only particle deformation but also particle rearrangement and void-volume reduction. Consequently, *D*_app_ and *λ*_app_ are not single-particle constitutive parameters or geometric pass/fail criteria. Because the same *ε*_b_ = 0.50 was applied to all three size fractions, *λ*_app_ is exactly one-half of the corresponding static λ and therefore preserves the original relative ordering of the hydrogel–core combinations. Thus, the identification of DHP-S as the least confined, DHP-L as the most strongly confined, and DHP-M as intermediate does not depend on assuming that Dapp is the true deformed diameter of an individual particle.

In particular, *λ*_app_ ≫ 1 indicates that free passage through the median throat is physically unlikely; any experimentally observed migration must instead involve selective access to relatively larger connected pathways together with deformation-mediated trapping, release, and re-migration.

As shown in Figure 6c, *λ*_app_ was lower than the corresponding static λ value for all hydrogel–core combinations. DHP-S showed *λ*_app_ values of 23.5, 42.9, and 81.0 in OTS-L, OTS-M, and OTS-H, respectively, indicating lower relative confinement within the tested particle series but potentially weaker retention. DHP-L exhibited much higher *λ*_app_ values of 90.6, 165.2, and 312.1, suggesting stronger bridging capacity but a higher risk of shallow plugging. DHP-M showed intermediate *λ*_app_ values of 47.8, 87.2, and 164.7, indicating a more balanced relationship between deformation-assisted migration and retention potential. Uncertainty propagation did not alter the relative confinement ranking among the tested hydrogel–core combinations; for DHP-M, the λapp values were 47.8 ± 6.1, 87.2 ± 11.4, and 164.7 ± 29.1 in OTS-L, OTS-M, and OTS-H, respectively.

The D10- and D90-based results further show that the particle size distribution produced a finite confinement envelope around the D50-based descriptor. For DHP-S, the *λ*_app_,10–*λ*_app_,90 ranges were 11.5–42.8, 21.0–78.0, and 39.6–147.4 in OTS-L, OTS-M, and OTS-H, respectively. The corresponding ranges were 24.9–81.8, 45.4–149.1, and 85.7–281.7 for DHP-M, and 49.0–149.4, 89.3–272.5, and 168.6–514.7 for DHP-L. These results indicate that the lower and upper tails of the particle size distribution experience different confinement levels, particularly near the empirical regime boundaries; therefore, the D50-based *λ*_app_ is retained as the central comparative descriptor, while the D10- and D90-based values define the lower and upper bounds of the particle size-dependent confinement range.

A sensitivity analysis was further performed to evaluate the influence of the prescribed bulk strain reference on λapp (Table S7). As εb increased from 20% to 50%, the calculated *λ*_app_ values decreased systematically, but the relative confinement ordering among the hydrogel–core combinations remained unchanged. For example, DHP-M in OTS-L, OTS-M, and OTS-H decreased from 76.5, 139.5, and 263.5 at 20% strain to 47.8, 87.2, and 164.7 at 50% strain, respectively. Combinations located close to the empirical boundaries showed greater sensitivity in their nominal regime assignment, further supporting the interpretation of *λ*_app_ = 25 and 80 as transition-oriented operational boundaries rather than sharp physical thresholds.

Figure 6d further classifies the hydrogel–core combinations into three operational confinement regimes. In the present dataset, *λ*_app_ = 25 and *λ*_app_ = 80 were used as empirical operational boundaries rather than universal critical values. The two boundaries were positioned between the low-, intermediate-, and high-confinement groups evident in the distribution of the tested hydrogel–core combinations in Figure 6d. Accordingly, *λ*_app_ < 25 was classified as a migration-dominated regime with relatively weak retention, 25 ≤ *λ*_app_ < 80 as an adaptive-migration regime with selective retention, and *λ*_app_ ≥ 80 as a high-confinement regime associated with premature-plugging risk.

The physical interpretation of these operational intervals was independently checked against the core-flooding responses. For DHP-M, the effluent particle recovery decreased from 42.8% in OTS-L to 24.0% in OTS-M and 8.6% in OTS-H, while the corresponding particle-retention efficiency increased from 58.0% to 78.0% and 91.4%, respectively. OTS-L therefore exhibited migration-dominated transport with relatively weak retention; OTS-M showed distributed retention extending beyond the inlet section; and OTS-H exhibited pronounced inlet-biased accumulation. These experimentally observed transitions are consistent with the three regimes represented in Figure 6d. Similar transitions from insufficient retention through favorable migration–retention matching to excessive confinement have also been reported in visualized elastic-gel-particle transport experiments [32].

The present coefficients should also be distinguished from conventional PPG particle-to-throat matching criteria. Previous studies commonly defined the matching coefficient as the ratio of particle diameter to a representative pore-throat diameter and reported favorable migration–retention behavior at ratios of approximately 0.20–0.32 for elastic gel particles and 0.21–0.29 for dispersed particle gels [32,33]. In contrast, the static *λ* values in the present tight sandstone system are much larger because the denominator is the MIP-derived median matrix-throat diameter, which is substantially smaller than the effective flow-channel dimensions typically used in sand-pack or visualized pore-throat models. Therefore, the numerical values of *λ* and *λ*_app_ are not intended to reproduce the established PPG matching ranges; rather, they describe the relative confinement of different hydrogel–core combinations within the present tight reservoir dataset. Nevertheless, both approaches show the same qualitative transition from insufficient retention at low relative confinement, through a migration–retention balance at intermediate confinement, to excessive interception or inlet-biased accumulation at high confinement.

More recently, Chen et al. showed that the transport behavior of branched preformed particle gels is jointly controlled by particle-to-throat matching, elastic modulus, and injection rate; decreasing the matching coefficient improved injectivity but reduced plugging efficiency [34]. This trend is qualitatively consistent with the present observation that weak confinement favors particle passage, whereas excessive confinement promotes stronger retention or localized accumulation. The numerical matching ranges cannot be directly compared because the two studies differ substantially in particle size, pore structure, mechanical properties, and the definition of the representative pore-throat scale.

Because the numerical boundaries depend on particle size and mechanics, pore-throat geometry and connectivity, fluid chemistry, and injection conditions, the values of 25 and 80 should be interpreted only as dataset-specific operational thresholds for the present hydrogel–core system and are not proposed as universal pore-throat matching criteria.

Taken together, these results support the selection of DHP-M for the subsequent transport and flow diversion experiments. Compared with DHP-S and DHP-L, DHP-M exhibited an intermediate apparent confinement level together with a balanced combination of hydrated size, bulk compressive response, and elastic recovery, providing a more suitable basis for migration and selective retention in the tested tight core systems.

### 2.5. Transport, Retention, and Pressure Response of Deformable Hydrogel Particles in Tight Cores

Based on the deformation-informed confinement analysis in Section 2.4, the core-scale transport experiments were organized into two complementary sets. First, DHP-M was evaluated in OTS-L, OTS-M, and OTS-H cores to determine how increasing pore-throat confinement affected particle transport and retention. Second, DHP-S, DHP-M, and DHP-L were directly compared in matched OTS-M cores under identical flooding conditions to isolate the influence of hydrogel particle size and experimentally verify the selection of DHP-M. Injection pressure, resistance factor, residual resistance factor, particle-retention efficiency, axial retention profile, and effluent particle recovery were analyzed to distinguish migration-dominated transport, distributed selective retention, and inlet-biased accumulation.

**Figure 7:**
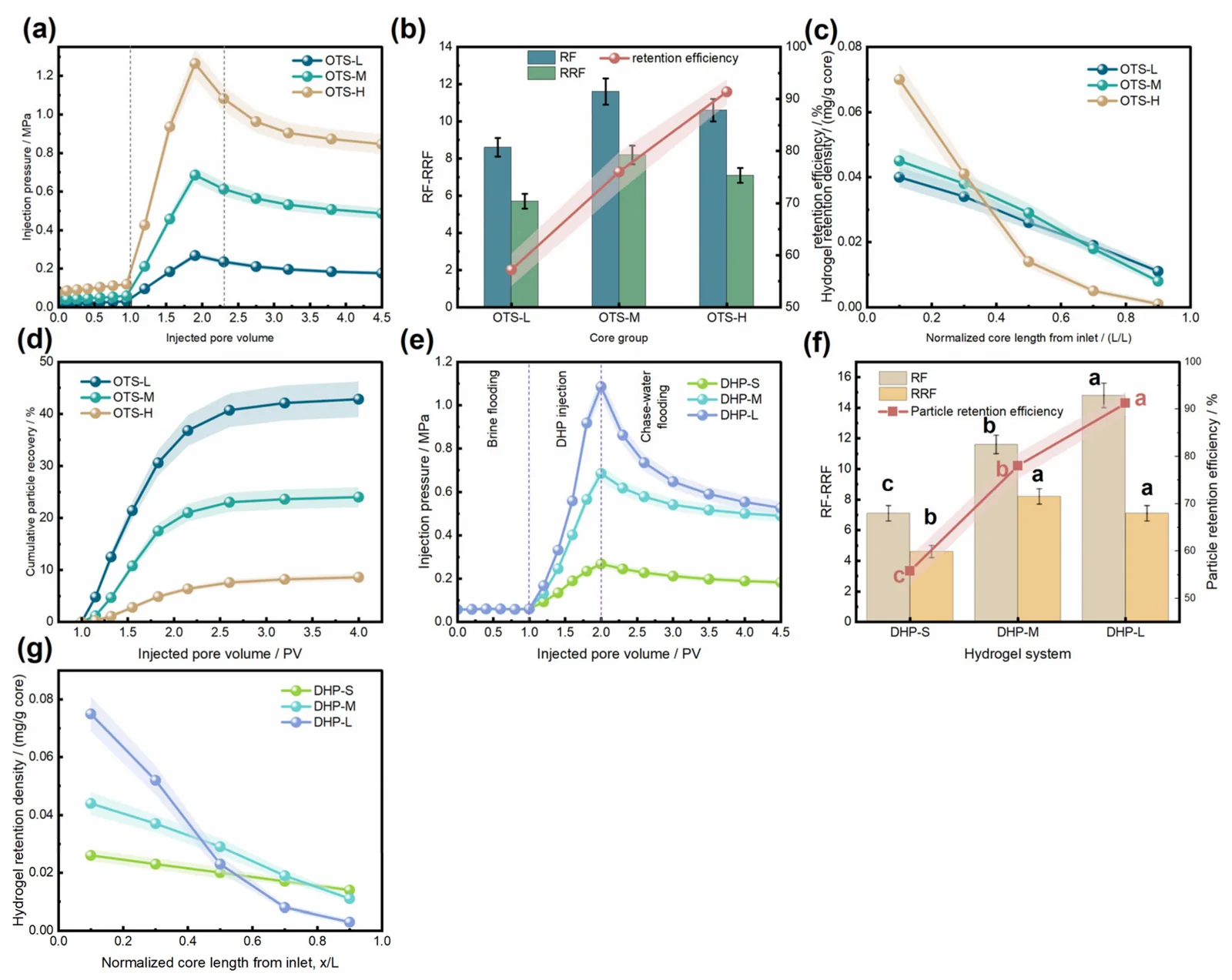
Transport and retention behavior of deformable hydrogel particles in Ordos tight cores: (**a**–**d**) transport behavior of DHP-M under different pore-throat confinement conditions, including (**a**) injection-pressure profiles during brine flooding, DHP-M injection, and chase-water flooding, (**b**) resistance factor, residual resistance factor, and particle-retention efficiency in OTS-L, OTS-M, and OTS-H cores, (**c**) axial hydrogel-retention density profiles along normalized core length, and (**d**) cumulative particle recovery from the effluent; (**e**–**g**) direct comparison of DHP-S, DHP-M, and DHP-L in matched OTS-M cores under identical flooding conditions, including (**e**) injection-pressure profiles, (**f**) resistance factor, residual resistance factor, and particle-retention efficiency, and (**g**) axial hydrogel-retention density profiles. Within each metric in panel (**f**), different lowercase letters indicate significant differences among DHP-S, DHP-M, and DHP-L according to Tukey’s HSD test (*p* < 0.05). The pressure profiles in panels (**a**,**e**) show representative individual core-flooding runs selected from three independent experiments for each condition and are not averaged curves. The summarized quantitative results in panels (**b**–**d**) and (**f**,**g**) are presented as mean ± standard deviation (*n* = 3), with error bars representing standard deviations. The vertical dashed lines in panels (**a**,**e**) delimit the hydrogel-particle injection stage.

As shown in Figure 7a, the pressure response varied markedly with pore-throat confinement. During initial brine flooding, the baseline pressures were 0.031, 0.059, and 0.119 MPa for OTS-L, OTS-M, and OTS-H, respectively. After DHP-M injection, the pressure increased to 0.268 MPa in OTS-L, 0.685 MPa in OTS-M, and 1.264 MPa in OTS-H. The gradual pressure increase in OTS-L indicates migration through relatively accessible connected pathways, whereas the rapid pressure buildup in OTS-H reflects stronger flow-pathway restriction and a higher probability of near-inlet accumulation. OTS-M exhibited an intermediate and controllable pressure response, consistent with selective transport followed by progressive retention within the connected pore-throat network.

The flow resistance parameters in Figure 7b show that the highest pressure did not correspond to the most favorable deep placement. The resistance factor increased from 8.6 in OTS-L to 11.6 in OTS-M, but decreased slightly to 10.6 in OTS-H. The residual resistance factor showed the same trend, with values of 5.7, 8.2, and 7.1, respectively. Particle retention efficiency increased from 58.0% in OTS-L to 78.0% in OTS-M and 91.4% in OTS-H, indicating stronger particle capture in tighter cores. However, the lower residual resistance factor (RRF) of OTS-H compared with OTS-M suggests that part of the retained particles contributed mainly to near-inlet resistance rather than distributed flow control.

The revised axial retention-density profiles in Figure 7c further distinguish the spatial placement of DHP-M. OTS-L showed relatively low but persistent retention toward the outlet, consistent with comparatively deep migration and limited overall capture. OTS-M maintained appreciable retention from the inlet into the middle region, producing a broader distributed retention zone rather than a sharply localized deposit. In contrast, OTS-H exhibited the highest inlet retention followed by a rapid axial decline, demonstrating pronounced inlet-biased accumulation. These profiles show that increasing confinement does not simply increase retention magnitude; it progressively shifts particle placement from deeper distributed retention toward localized near-inlet capture.

The cumulative effluent particle recovery in Figure 7d was consistent with these spatial differences. Particle recovery decreased from 42.8% in OTS-L to 24.0% in OTS-M and 8.6% in OTS-H. The relatively high recovery from OTS-L is consistent with migration through more accessible connected pathways, whereas the limited recovery from OTS-H reflects strong particle capture under severe confinement. Together with the axial retention profiles, the breakthrough behavior identifies a transition from migration-dominated transport in OTS-L, through adaptive migration with distributed selective retention in OTS-M, to confinement-controlled inlet accumulation in OTS-H.

To compare the transport behavior of the three emulsification-speed-generated particle size classes under the same pore-throat environment, DHP-S, DHP-M, and DHP-L were evaluated using matched OTS-M cores. As shown in Figure 7e, the three systems had similar baseline pressure drops before particle injection. The peak injection pressure increased from 0.42 MPa for DHP-S to 0.69 MPa for DHP-M and 0.87 MPa for DHP-L. The lower pressure response of DHP-S reflects weaker restriction during transport, whereas the pronounced pressure buildup of DHP-L indicates stronger confinement and earlier particle accumulation. DHP-M showed an intermediate pressure response, suggesting that appreciable flow resistance developed without excessive pressure buildup.

The flow-resistance parameters in Figure 7f further distinguish the three transport behaviors. Resistance factor (RF) increased from 7.1 for DHP-S to 11.6 for DHP-M and 14.8 for DHP-L, while particle-retention efficiency increased from 55.8% to 78.0% and 91.2%, respectively. In contrast, RRF increased from 4.6 for DHP-S to a numerical maximum of 8.2 for DHP-M and then decreased to 7.1 for DHP-L. The overall group effect was significant (F(2,6) = 46.409, *p* = 0.000224). Tukey’s HSD test showed significant differences between DHP-S and DHP-M (*p* = 0.000203) and between DHP-S and DHP-L (*p* = 0.001501), whereas the difference between DHP-M and DHP-L was not statistically significant (*p* = 0.063768; 95% simultaneous CI for the mean difference: −0.08 to 2.28). This non-monotonic response indicates that stronger particle capture does not necessarily generate more effective residual flow resistance. The high retention but lower RRF of DHP-L is consistent with localized accumulation, whereas DHP-M maintained stronger post-treatment flow resistance through more spatially distributed particle placement.

The revised axial retention-density profiles in Figure 7g provide direct spatial support for this interpretation. DHP-S maintained the lowest retention level but showed the slowest axial decay, indicating greater penetration depth with insufficient particle placement. DHP-L exhibited pronounced inlet enrichment followed by rapid attenuation toward the outlet, consistent with premature localized capture. DHP-M showed an intermediate inlet retention level while maintaining appreciable retention through the middle portion of the core, producing a broader effective placement zone. This spatial distribution explains why DHP-M did not maximize either injectability or total particle capture individually, but instead provided the most favorable balance among migration depth, distributed retention, and persistent flow resistance under the tested OTS-M conditions.

These results support the use of DHP-M in the subsequent heterogeneous flow-redistribution experiments. A focused rate-sensitivity test in matched OTS-M cores further showed that increasing the injection rate from 0.10 to 0.30 mL/min increased the peak apparent pressure gradient from 9.2 to 18.2 MPa/m, decreased particle-retention efficiency from 85.4% to 69.0%, and increased cumulative effluent recovery from 16.1% to 32.6% (Table S5). This response indicates that a higher hydrodynamic driving force promoted deformation-assisted passage and re-migration, while the standard rate of 0.20 mL/min provided an intermediate balance between transport and retention. A similar injection-rate dependence has been reported by Tang et al. [35], who used low-field NMR to show that injection rate affected the penetration and plugging behavior of branched preformed particle gels in fractured cores. Although the experimental geometry differs from the present tight sandstone matrix system, both studies indicate that hydrodynamic forcing is an important factor governing the balance between particle transport and retention.

### 2.6. Flow Redistribution Performance in Heterogeneous Parallel Core Systems

The single-core transport results showed that DHP-M could form different retention patterns depending on pore-throat confinement. To examine whether this retention could regulate flow distribution in heterogeneous systems, parallel core models were constructed using OTS-L, OTS-M, and OTS-H cores. The three heterogeneous core systems (HCSs), HCS-LM (OTS-L/OTS-M), HCS-LH (OTS-L/OTS-H), and HCS-MH (OTS-M/OTS-H), represented mild, strong, and intermediate-to-strong heterogeneity, respectively.

**Figure 8:**
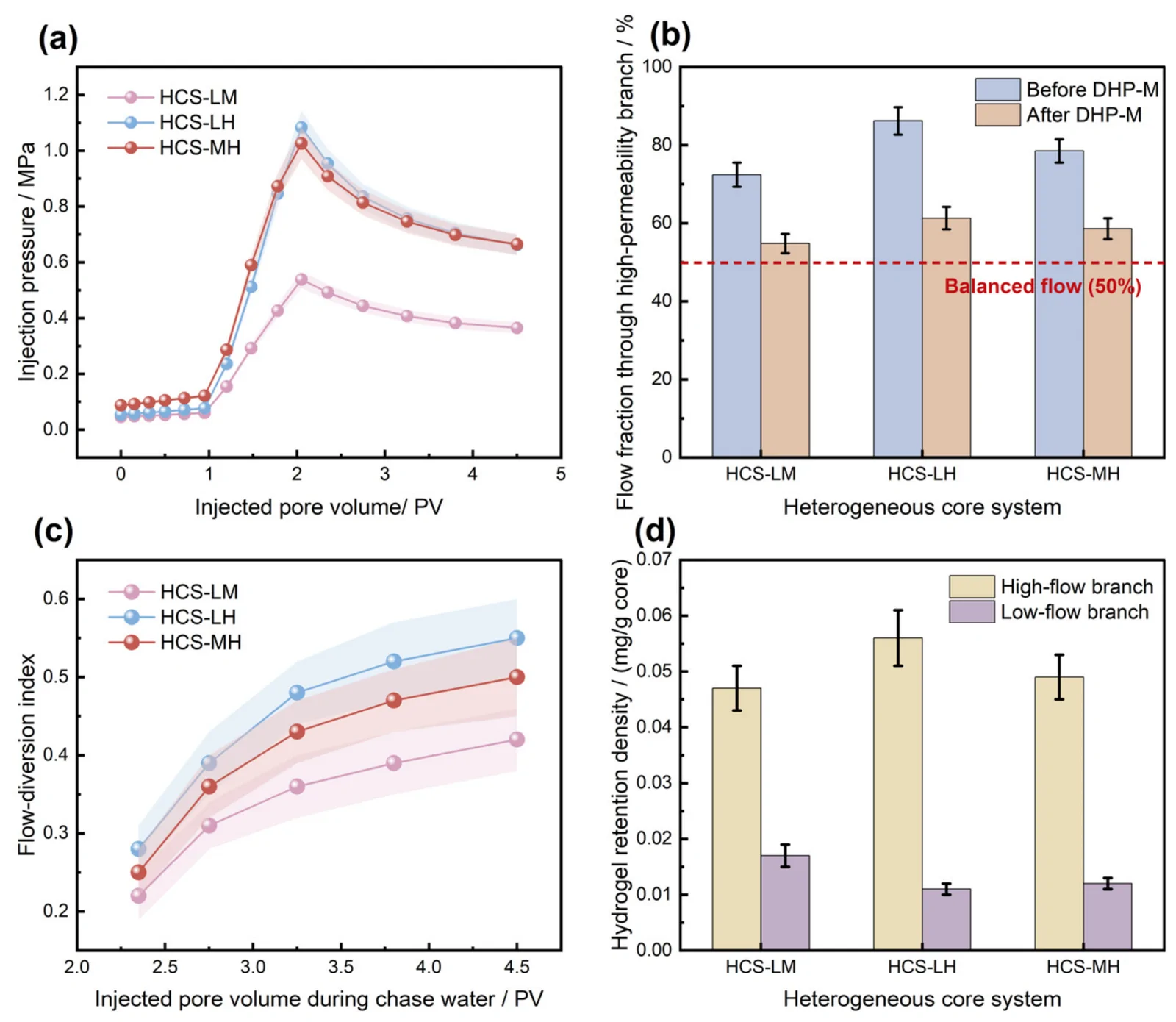
Flow-redistribution behavior of DHP-M in heterogeneous parallel core systems: (**a**) injection pressure profiles during brine flooding, DHP-M injection, and chase-water flooding; (**b**) flow fraction through the high-permeability branch before and after DHP-M treatment; (**c**) flow diversion index during chase-water flooding; (**d**) dry hydrogel retention density normalized by dry core mass in the high-flow and low-flow branches. The pressure profiles in panel (**a**) show representative individual runs from the three independently assembled parallel core systems for each condition and are not averaged curves. The summarized quantitative results in panels (**b**–**d**) are presented as mean ± standard deviation (*n* = 3), with error bars representing standard deviations.

As shown in Figure 8a, DHP-M injection generated clear pressure-building responses in all heterogeneous systems. In HCS-LM, the pressure increased from 0.060 MPa to 0.538 MPa during DHP-M injection and then decreased to 0.365 MPa during chase-water flooding. HCS-LH showed a stronger pressure response, increasing from 0.077 MPa to 1.083 MPa, while HCS-MH reached a similar peak pressure of 1.026 MPa. The higher pressure buildup in HCS-LH and HCS-MH indicates stronger particle retention in the dominant flow branch under larger permeability contrast.

For field-injectivity assessment, the laboratory pressure response was further compared with reported fracture pressure conditions in Ordos tight reservoirs. Based on the 50 mm core length, the peak pressure drops measured in the single-core experiments correspond to apparent laboratory pressure gradients of approximately 5.36–25.28 MPa/m, while those in the heterogeneous parallel core systems correspond to approximately 10.76–21.66 MPa/m. These values are much higher than field-scale fracture pressure gradients because the laboratory gradient is normalized over an extremely short core length and therefore should not be directly extrapolated to reservoir scale. For a representative tight sandstone gas reservoir in the Ordos Basin, a fracture pressure gradient of approximately 0.018–0.022 MPa/m has been reported at a burial depth of about 2330 m, corresponding to a fracture pressure range of approximately 42–51 MPa, compared with a formation pressure of about 20.5 MPa [36]. Thus, the more relevant comparison is the absolute additional pressure drop generated by hydrogel placement. The maximum laboratory pressure increment remained below approximately 1.3 MPa, which is substantially smaller than the reported field-scale pressure margin between formation pressure and fracture pressure. This comparison suggests that DHP-M may be injectable without approaching fracture initiation under appropriately controlled field injection conditions. However, field-scale injection pressure should ultimately be constrained by well-specific fracture pressure measurements, such as mini-frac or diagnostic fracture-injection tests, rather than by direct scaling of the laboratory pressure gradient.

The change in branch flow fraction further confirms the flow diversion effect. As shown in Figure 8b, before DHP-M treatment, the high-flow branch carried 72.4 ± 3.1%, 86.2 ± 3.5%, and 78.5 ± 3.0% of the total flow in HCS-LM, HCS-LH, and HCS-MH, respectively. After treatment, these values decreased to 54.8 ± 2.5%, 61.3 ± 2.9%, and 58.6 ± 2.7%. The decrease in high-flow branch fraction indicates that DHP-M selectively reduced the conductivity of preferential pathways. The flow distribution approached, but did not fully reach, the balanced-flow state, reflecting the remaining hydraulic resistance in the low-flow branch.

The flow diversion index in Figure 8c describes the persistence of this redistribution during chase-water flooding. The final index reached 0.42 ± 0.04 for HCS-LM, 0.55 ± 0.05 for HCS-LH, and 0.50 ± 0.05 for HCS-MH. HCS-LH showed the highest value because its stronger initial heterogeneity provided a more distinct preferential pathway for DHP-M entry and retention. By contrast, HCS-LM had a lower diversion index because its initial flow distribution was less imbalanced, leaving less room for further redistribution.

The branch-specific retention densities in Figure 8d provide direct evidence of preferential particle placement. In all three heterogeneous systems, DHP-M retention was substantially higher in the high-flow branch than in the corresponding low-flow branch, confirming preferential entry and capture within the hydraulically dominant pathway. The contrast was particularly pronounced in HCS-LH, where the stronger initial permeability contrast promoted selective particle transport toward the high-conductivity branch. The retention pattern therefore agrees with the observed reduction in high-flow fraction and the persistence of flow diversion during subsequent water flooding.

The combined pressure, flow-fraction, diversion-index, and retention-density results indicate that DHP-M did not uniformly plug the parallel core system. Instead, it preferentially reduced the conductivity of the high-flow branch and redirected subsequent water toward the low-flow branch. This behavior differs from simple inlet-end blockage because the flow regulation was associated with selective particle placement in the dominant pathway. These parallel core experiments therefore provide direct evidence of branch-scale selective retention and flow redistribution under controlled heterogeneity. However, the two-branch configuration does not reproduce the spatial continuity, scale, multidirectional connectivity, or fracture–matrix interactions of an actual reservoir. Accordingly, the present results should be interpreted as demonstrating the potential mechanism for deep flow diversion rather than as direct evidence of reservoir-scale deep placement. Validation in longer continuous cores or larger two- or three-dimensional heterogeneous reservoir models is required to establish the effective diversion distance under field-relevant conditions. The resulting redistribution provides the experimental basis for evaluating movable fluid activation and displacement enhancement in Section 2.7.

### 2.7. Movable Fluid Redistribution and Displacement Enhancement After DHP-M Treatment

The flow diversion results in Section 2.6 showed that DHP-M preferentially reduced the conductivity of the high-flow branch. To further determine whether this redistribution activated under-swept pore-throat domains, movable fluid saturation, branch flow fraction, cumulative oil recovery, and recovery increment were evaluated after DHP-M-assisted chase-water flooding.

**Figure 9:**
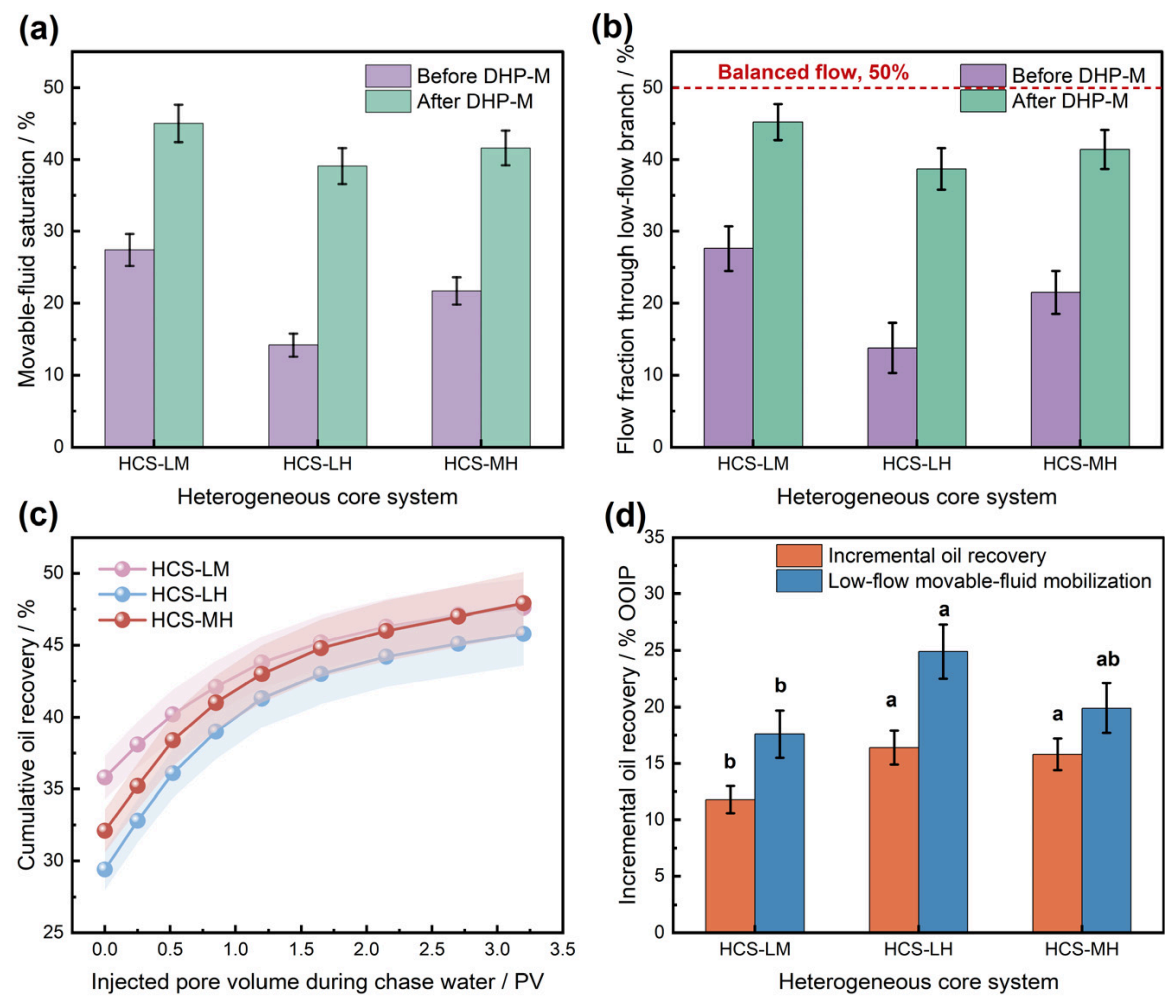
Movable fluid redistribution and displacement enhancement after DHP-M-assisted flow diversion: (**a**) movable fluid saturation in the low-flow branch before and after DHP-M treatment; (**b**) flow fraction through the low-flow branch before and after DHP-M treatment; (**c**) cumulative oil recovery during chase-water flooding; (**d**) incremental oil recovery and low-flow movable fluid mobilization increment. Different lowercase letters indicate significant differences among heterogeneous core systems within the same metric (Tukey’s HSD, *p* < 0.05). Data are expressed as mean ± standard deviation from three independently assembled parallel core systems (*n* = 3). Incremental oil recovery in panel (**d**) denotes the absolute increase in original oil in place (OOIP)-based recovery factor after DHP-M treatment and is expressed in percentage points, not as a relative percentage increase from the water-flooding recovery.

As shown in Figure 9a, the NMR-derived movable fluid saturation in the low-flow branch increased in all heterogeneous systems after DHP-M treatment. The value increased from 27.4 ± 2.2% to 45.0 ± 2.6% in HCS-LM, from 14.2 ± 1.6% to 39.1 ± 2.5% in HCS-LH, and from 21.7 ± 1.9% to 41.6 ± 2.4% in HCS-MH. The largest increase occurred in HCS-LH, suggesting that the strongly heterogeneous system retained more movable fluid in initially under-swept domains.

The branch-flow distribution showed a consistent trend. As shown in Figure 9b, the flow fraction through the low-flow branch increased from 27.6 ± 3.1% to 45.2 ± 2.5% in HCS-LM, from 13.8 ± 3.5% to 38.7 ± 2.9% in HCS-LH, and from 21.5 ± 3.0% to 41.4 ± 2.7% in HCS-MH. The treated systems approached the balanced-flow line but did not reach complete equalization, reflecting the remaining pore-throat restriction in the low-flow branch. Although Figure 9a,b show similar trends, they represent different measurements: movable fluid saturation was derived from NMR fluid distribution, whereas branch flow fraction was calculated from effluent volumes.

The cumulative oil recovery curves in Figure 9c further show that flow redistribution contributed to displacement enhancement. Oil recovery increased from 35.8 ± 1.5% to 47.6 ± 2.0% in HCS-LM, from 29.4 ± 1.4% to 45.8 ± 2.2% in HCS-LH, and from 32.1 ± 1.5% to 47.9 ± 2.2% in HCS-MH. These improvements indicate that DHP-M treatment redirected chase water into previously under-swept regions rather than merely increasing the injection pressure. This coupling between preferential-pathway restriction, redistribution of subsequent water, and enhanced recovery is consistent with recent PPG studies in heterogeneous flow systems. Seidy-Esfahlan et al. [37] demonstrated in dual parallel sandpack experiments that PPG treatment reduced preferential-pathway conductivity and promoted flow redistribution toward lower-permeability regions, thereby improving sweep efficiency and oil recovery. Although the porous-medium geometry and gel formulation differ from those used in the present study, the reported flow-redistribution behavior is qualitatively consistent with the simultaneous changes in branch flow fraction, NMR-derived movable fluid saturation, and OOIP-based recovery factor observed here. Together with the matched brine-only controls, these results support the interpretation that the recovery improvement was associated with DHP-M-induced flow redistribution rather than continued water flooding alone.

As summarized in Figure 9d, DHP-M treatment increased the OOIP-based recovery factor by 11.8, 16.4, and 15.8 percentage points in HCS-LM, HCS-LH, and HCS-MH, respectively. The corresponding low-flow movable fluid mobilization increments were 17.6 ± 2.1%, 24.9 ± 2.4%, and 19.9 ± 2.2%. The stronger response in HCS-LH indicates that DHP-M-assisted diversion was more effective when a distinct preferential pathway existed and the low-flow region still contained recoverable movable fluid.

To distinguish the DHP-M contribution from continued water flooding and fluid redistribution during shut-in, matched brine-only controls were evaluated under the same operating conditions. As summarized in Table S3, the untreated systems showed only limited changes in the low-flow branch, with movable fluid saturation increasing by 2.5–3.2 percentage points, low-flow branch fraction increasing by 2.2–2.9 percentage points, and the OOIP-based recovery factor increasing by less than 2.5 percentage points. In contrast, DHP-M treatment increased low-flow movable fluid saturation by 17.6–24.9 percentage points and increased the OOIP-based recovery factor by 11.8–16.4 percentage points. The substantially larger response after DHP-M treatment indicates that continued water flooding and fluid redistribution during shut-in alone cannot account for the observed flow redistribution and displacement enhancement.

These results show that DHP-M improved displacement performance by first reducing the conductivity of the dominant flow branch and then redirecting chase water into less-swept pore-throat domains. The simultaneous increases in low-flow branch flow fraction, movable fluid saturation, and oil recovery support its role as a pore-throat-adaptive flow diversion material rather than a simple near-inlet plugging agent.

### 2.8. Mechanism of Pore-Throat-Adaptive Transport and Flow Redistribution

**Figure 10:**
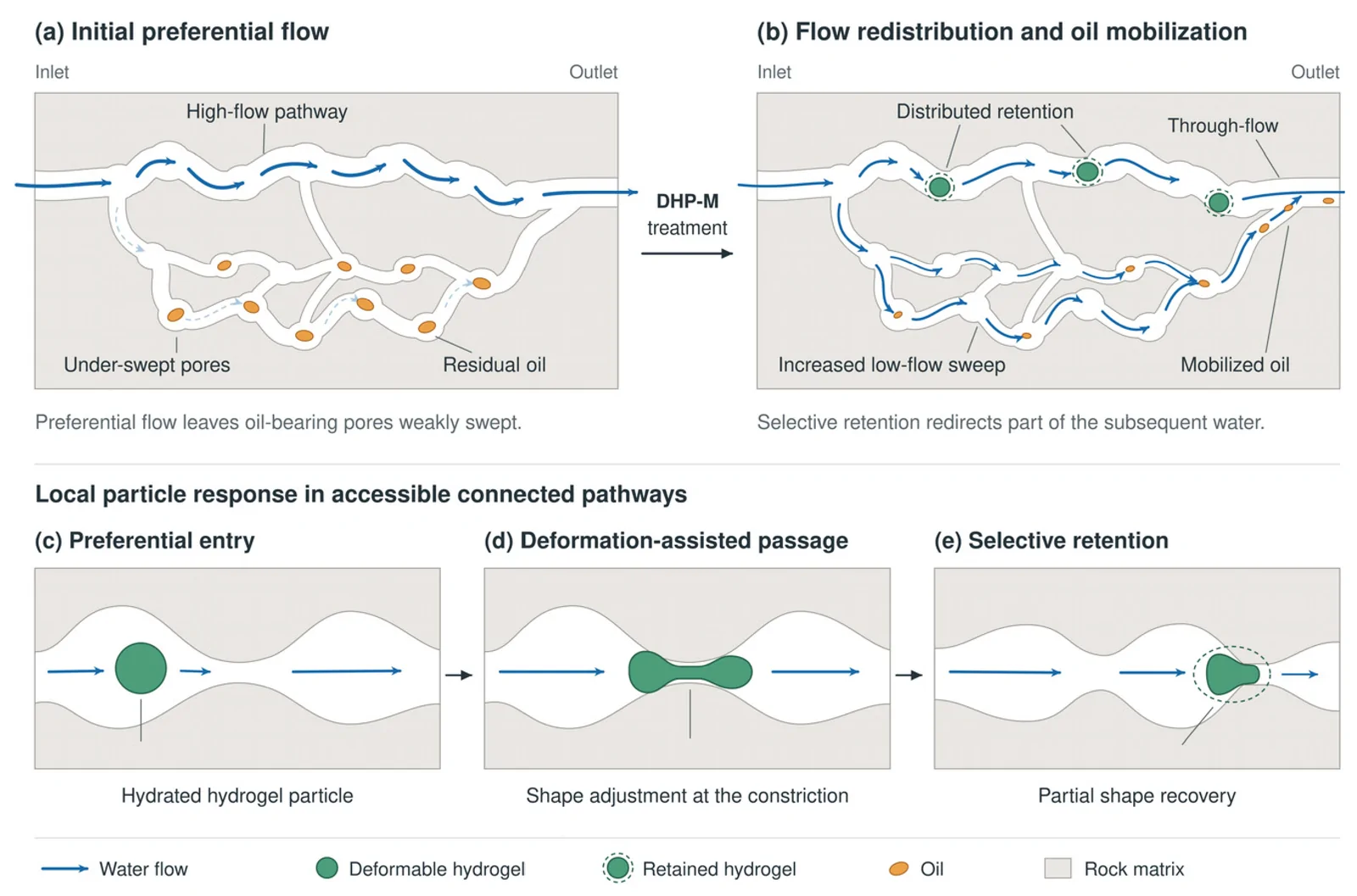
Conceptual schematic of the proposed pore-throat-adaptive transport and flow-redistribution mechanism of deformable hydrogel particles in Ordos tight cores: (**a**) initial preferential flow; (**b**) flow redistribution and oil mobilization; (**c**) preferential entry; (**d**) deformation-assisted passage; and (**e**) selective retention following partial shape recovery. he dotted blue arrow indicates weak water flow into the under-swept pores before treatment. Schematic, not to scale.

The results above show that the transport of DHP-M in tight cores cannot be described by a simple rigid-particle sieving model. Although the hydrated particle size was much larger than the MIP-derived median characteristic throat diameter, core-flooding experiments nevertheless showed partial particle breakthrough and spatially distributed retention. This observation should not be interpreted as direct passage of intact DHP-M particles through the submicron median throats. Rather, the combined pressure response, particle breakthrough, axial retention, compression–recovery behavior, and post-flood imaging results are consistent with DHP-M transport through relatively accessible connected pathways accompanied by deformation and temporary retention at local constrictions. However, the temporal sequence of particle deformation, passage, shape recovery, and re-migration was not directly visualized and is therefore interpreted as a proposed transport scenario rather than a directly observed process. Therefore, its flow diversion behavior was governed by the combined effects of hydrated size, deformation recovery, throat-size distribution, pore-network connectivity, and initial movable fluid distribution. The proposed mechanism is illustrated in Figure 10.

Before DHP-M injection, the tight cores showed distinct pore-throat accessibility. OTS-L contained relatively developed connected pathways and higher movable fluid saturation, while OTS-H was dominated by finer throats, weaker connectivity, and a larger fraction of bound fluid. OTS-M represented an intermediate pore-throat environment, where connected pathways and local throat constrictions coexisted. This structural sequence explains why injected water preferentially flowed through more accessible channels and left tighter pore-throat domains insufficiently swept.

After hydration, DHP-M exhibited an intermediate particle size and recoverable deformation, allowing it to enter connected pathways under pressure and deform at local throat constrictions. In OTS-L, the particles migrated more deeply but showed weaker retention because the pore network was more open. In OTS-H, particle capture was stronger, but retention was concentrated near the inlet because narrow throats limited further penetration. In OTS-M, DHP-M showed the most balanced behavior: it migrated beyond the inlet region and was progressively retained along connected pore-throat pathways. This distributed retention was more favorable for flow control than local inlet plugging.

Quantitative analysis of the registered micro-CT volumes further showed that DHP-M retention altered the resolvable connected pore network to different extents among the three core groups (Table S4). Connected porosity decreased from 7.26% to 6.95% in OTS-L, from 4.42% to 3.84% in OTS-M, and from 1.86% to 1.74% in OTS-H. The corresponding geometric tortuosity increased from 1.41 to 1.47, 1.69 to 1.95, and 2.12 to 2.23, respectively. The largest relative decrease in connected porosity (13.1%) and increase in tortuosity (15.4%) occurred in OTS-M, consistent with its broader distributed retention zone and higher residual resistance. By contrast, the smaller changes in OTS-L reflect weaker particle retention, whereas the limited network-scale change in OTS-H is consistent with predominantly inlet-biased accumulation. These results indicate that effective flow regulation depends not simply on maximizing particle retention, but on modifying the continuity and complexity of connected flow pathways.

In heterogeneous core systems, DHP-M preferentially entered the high-flow branch because of its lower hydraulic resistance. The retained particles reduced the conductivity of this dominant pathway and redirected chase water toward the low-flow branch. The increase in low-flow branch flow fraction, movable fluid saturation, and cumulative oil recovery indicates that flow redistribution activated previously under-swept pore-throat domains rather than merely increasing injection pressure.

Taken together, the experimental results support a conceptual transport pathway involving preferential entry, deformation-assisted transport, selective retention, and subsequent flow redistribution. The specific sequence of individual-particle deformation, recovery, and re-migration remains mechanistically inferred from the complementary bulk and post-flood measurements rather than directly resolved in situ. Particles that are too small may pass through connected channels with limited retention, whereas particles that are too large may accumulate near the inlet and restrict deep placement. DHP-M provided a more suitable balance between entry, deformation, recovery, and retention, enabling pore-throat-adaptive transport and deep flow diversion in heterogeneous tight cores.

### 2.9. Limitations

Several limitations should be considered when extrapolating the present results. The 50 mm-long core plugs capture core-scale transport and retention but cannot reproduce the propagation distance and pressure evolution expected over reservoir-scale flow paths. In addition, the parallel core models represent heterogeneity through a simplified two-branch permeability contrast and do not reproduce the spatial continuity, multidirectional connectivity, or fracture–matrix interactions of naturally heterogeneous reservoirs. The laboratory flooding and hydration periods are also substantially shorter than field treatment and production timescales, and explicit transport through field-scale fracture networks was not evaluated. Therefore, the present results should be interpreted as evidence for the feasibility of pore-throat-adaptive transport and selective flow redistribution under controlled laboratory conditions rather than as direct prediction of field-scale placement or treatment performance.

## 3. Conclusions

AM/AMPS/AA-based deformable hydrogel particles with tunable particle size and network structure were prepared by inverse emulsion polymerization. Emulsification conditions primarily regulated particle size, whereas crosslinking density controlled swelling and deformation behavior. Among the tested formulations, DHP-M provided the most suitable balance between hydrated size, compressive response, and recovery for the investigated tight sandstone pore-throat conditions.

The deformation-informed matching analysis and core-flooding experiments showed that increasing pore-throat confinement shifted particle behavior from migration-dominated transport to distributed retention and then to inlet-biased accumulation. DHP-M exhibited favorable distributed retention under intermediate confinement, supporting a transport mechanism governed by the combined effects of particle size distribution, deformability, and pore-network accessibility rather than simple rigid-particle sieving.

In heterogeneous parallel core systems, DHP-M preferentially reduced conductivity in the high-flow branch and redistributed subsequent water toward the low-flow branch. This redistribution increased the OOIP-based recovery factor by 11.8–16.4 percentage points under the tested conditions. These results provide a laboratory-scale basis for designing deformable hydrogel particles through coordinated control of particle size, mechanical adaptability, and pore-throat confinement.

## 4. Materials and Methods

### 4.1. Experimental Materials

The materials used in this study are summarized in Table 7. Acrylamide (AM), 2-acrylamido-2-methylpropane sulfonic acid (AMPS), and acrylic acid (AA) were used as the main monomers for constructing deformable hydrogel particles. N,N′-methylenebisacrylamide (MBAA) was used as the crosslinking agent, while ammonium persulfate (APS) and sodium bisulfite (NaHSO_3_) were used as the redox initiation system. Liquid paraffin, Span 80, and Tween 80 were used to form the inverse emulsion system. Sodium chloride (NaCl), calcium chloride (CaCl_2_), and magnesium chloride hexahydrate (MgCl_2_·6H_2_O) were used to prepare simulated formation water. Natural tight sandstone cores were obtained from the Ordos Basin and used for pore-throat characterization, injectability evaluation, transport-retention analysis, and deep flow diversion experiments. All chemicals were used as received without further purification unless otherwise specified.

### 4.2. Experimental Instruments

The major experimental instruments and testing systems used in this study are summarized in Table 8. These instruments were selected to characterize the chemical structure, particle morphology, particle size distribution, swelling behavior, rheological properties, compression recovery, pore-throat structure, core-scale transport, and flow diversion behavior of deformable hydrogel particles in tight reservoir cores.

### 4.3. Preparation of Deformable Hydrogel Particles

**Figure 11:**
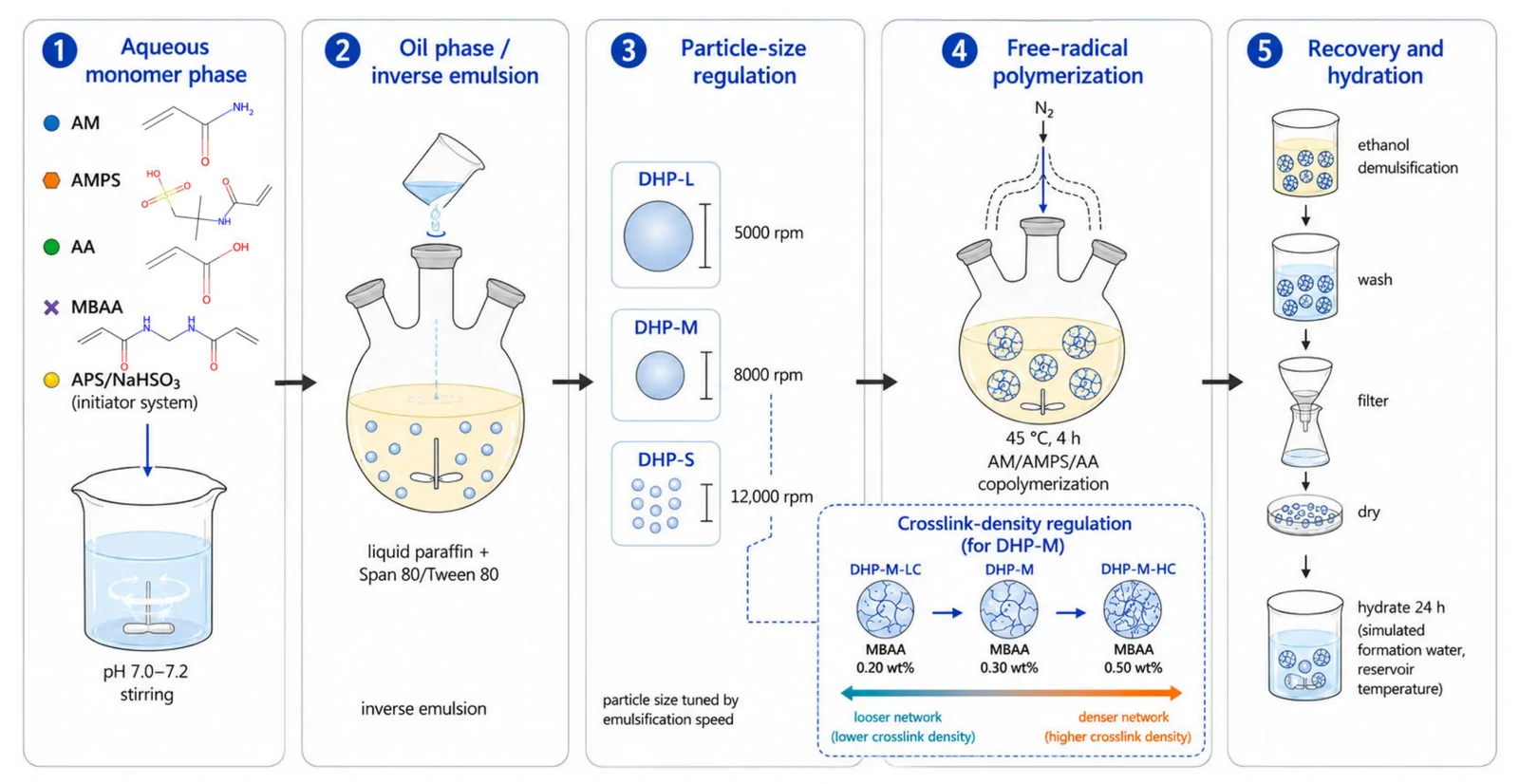
Schematic illustration of the preparation process and structural design of pore-throat-adaptive deformable hydrogel particles. Black arrows indicate the preparation sequence, while colored arrows indicate the direction of crosslink-density regulation. Colored symbols in step 1 identify the corresponding reagents. Spheres illustrate relative particle sizes, and mesh-patterned circles represent polymer network structures.

Deformable hydrogel particles were prepared by inverse emulsion free-radical polymerization. Acrylamide (AM), 2-acrylamido-2-methylpropane sulfonic acid (AMPS), and acrylic acid (AA) were used as monomers, N,N′-methylenebisacrylamide (MBAA) was used as the crosslinking agent, and ammonium persulfate (APS)/sodium bisulfite (NaHSO_3_) was used as the redox initiation system. The preparation process is illustrated in Figure 11.

For the aqueous phase, 60.0 mL of deionized water was added to a 250 mL beaker and stirred at 300 rpm for 10 min. AM, AMPS, and AA were then dissolved sequentially at masses of 4.20, 1.20, and 0.60 g, respectively. The pH was adjusted to 7.0–7.2 using 1.0 mol/L NaOH solution. After complete dissolution, MBAA was added according to the dosages listed in Table 9, and the solution was stirred at 300 rpm for 20 min.

For the oil phase, 120.0 mL of liquid paraffin was added to a 500 mL three-necked flask, followed by 3.00 g of Span 80 and 0.60 g of Tween 80. The mixture was stirred at 500 rpm for 20 min. The aqueous phase was then slowly added dropwise to the oil phase under stirring, followed by emulsification with a high-speed homogenizer for 10 min. Emulsification speeds of 12,000, 8000, and 5000 rpm were used to prepare DHP-S, DHP-M, and DHP-L, respectively, with the same MBAA dosage of 0.018 g.

After emulsification, the system was mechanically stirred at 500 rpm and purged with nitrogen for 20 min. APS and NaHSO_3_ were separately dissolved in small amounts of deionized water and sequentially added to the emulsion. The dosages of APS and NaHSO_3_ were both 0.030 g. The reaction temperature was then increased to 45 °C and maintained for 4 h under continuous stirring.

Two additional medium-sized samples with different crosslinking densities were prepared using the same procedure as DHP-M. The MBAA dosages were 0.012 g for DHP-M-LC and 0.030 g for DHP-M-HC, corresponding to 0.20 wt% and 0.50 wt% of the total monomer mass, respectively. The standard DHP-M sample contained 0.018 g MBAA, corresponding to 0.30 wt% of the total monomer mass. To distinguish the roles of emulsification conditions and network crosslinking, the five hydrogel formulations were organized into two controlled experimental series. In the first series (DHP-S, DHP-M, and DHP-L), the monomer composition and MBAA dosage were fixed at 0.30 wt%, while only the emulsification speed was varied at 12,000, 8000, and 5000 rpm, respectively. In this series, emulsification speed was treated as the manipulated synthesis variable, whereas dry and hydrated particle sizes were measured as structural responses rather than independent preparation factors. The resulting particle size classes were subsequently compared under identical core-flooding conditions to evaluate their transport and retention behavior. In the second series (DHP-M-LC, DHP-M, and DHP-M-HC), the emulsification speed was fixed at 8000 rpm and the monomer composition remained unchanged, while the MBAA dosage was varied from 0.20 to 0.50 wt%. Because the dry D50 values of these samples remained within a narrow range of 71.6–74.1 μm, differences in swelling, hydrated size, network morphology, compression response, and elastic recovery were interpreted primarily in terms of crosslinking-density variation. The two experimental series were evaluated separately, and no interaction between emulsification speed and MBAA dosage was inferred from the present design.

After polymerization, the emulsion was cooled to room temperature and demulsified by adding 200 mL of anhydrous ethanol under stirring at 300 rpm for 20 min. The hydrogel particles were separated by vacuum filtration and washed three times with petroleum ether and ethanol to remove residual oil phase, emulsifiers, and soluble impurities. The collected particles were dried in a vacuum oven at 45 °C for 24 h, gently ground to break weak agglomerates, and stored in sealed containers.

Before characterization and flooding tests, the dried hydrogel particles were hydrated in simulated formation water at 60 °C for 24 h. For core transport and heterogeneous flow diversion experiments, a 0.30 wt% hydrogel particle suspension was prepared and magnetically stirred at 300 rpm for 30 min before injection.

### 4.4. Preparation of Simulated Formation Water and Hydrogel Particle Dispersions

Simulated formation water was prepared using NaCl, CaCl_2_, and MgCl_2_·6H_2_O. Unless otherwise specified, the total salinity was 100,000 mg/L, with NaCl, CaCl_2_, and MgCl_2_·6H_2_O concentrations of 80,000, 12,000, and 8000 mg/L, respectively. Based on reagent stoichiometry, the corresponding nominal concentrations of the major dissolved ions were approximately 31,470 mg/L Na^+^, 4333 mg/L Ca^2+^, 956 mg/L Mg^2+^, and 58,987 mg/L Cl^−^. These values represent the nominal ionic composition calculated from the added salts rather than independently measured ion concentrations.

For salinity-response tests, brines with total salinities of 50,000, 100,000, and 150,000 mg/L were prepared by proportionally adjusting the concentrations of all three salts while maintaining the same NaCl:CaCl_2_:MgCl_2_·6H_2_O mass ratio of 80:12:8. Accordingly, the corresponding NaCl/CaCl_2_/MgCl_2_·6H_2_O concentrations were 40,000/6000/4000 mg/L, 80,000/12,000/8000 mg/L, and 120,000/18,000/12,000 mg/L, respectively.

All brines were prepared with deionized water, stirred at 500 rpm for 30 min, and filtered through a 0.45 μm membrane before use. The pH of the freshly prepared standard 100,000 mg/L simulated formation water was 6.4 ± 0.1 at 25 °C, as determined using a calibrated PHS-3C pH meter.

The simulated brine was designed as a simplified high-salinity, chloride-dominated aqueous system rather than an exact reproduction of formation water from a specific Ordos well. Formation waters from tight sandstone reservoirs in the Ordos Basin are generally characterized by Cl^−^-dominated anionic compositions, Na^+^ and Ca^2+^ as major cations, CaCl_2_-type water chemistry, and a broad salinity range. Reported formation-water pH values are generally weakly acidic, commonly ranging from approximately 5.5 to 7.1. Therefore, the 100,000 mg/L brine with a pH of approximately 6.4 was selected to represent the high-ionic-strength and weakly acidic aqueous environment relevant to Ordos tight reservoirs.

Because actual Ordos formation waters also contain variable amounts of K^+^, HCO_3_^−^, SO_4_^2−^, Sr^2+^, Ba^2+^, and other minor ions, the present NaCl–CaCl_2_–MgCl_2_ system should be regarded as a controlled laboratory analog that captures the principal salinity and major-ion effects rather than the complete geochemical composition of field formation water.

Before particle size measurement, swelling tests, and core-flooding experiments, the dried hydrogel particles were hydrated in simulated formation water. Typically, 0.10 g of dried particles was added to 100 mL of simulated formation water and kept at 60 °C for 24 h to reach swelling equilibrium. During hydration, the suspension was gently shaken at regular intervals to reduce particle settling. Strong ultrasonication was not used to avoid damaging the weakly crosslinked hydrogel particles or altering their original size distribution.

For core transport and flow diversion experiments, hydrated hydrogel particle dispersions with a concentration of 0.30 wt% were prepared using 100,000 mg/L simulated formation water. The hydrated particles were dispersed by magnetic stirring at 300 rpm for 30 min before injection. The dispersion was used immediately after preparation to minimize sedimentation and concentration fluctuation during core flooding. The rheological behavior of 0.30 wt% DHP-M dispersions was measured using an MCR 302 rotational rheometer at 60 °C over a shear-rate range of 1–100 s^−1^ in brines with salinities of 50,000, 100,000, and 150,000 mg/L; apparent viscosities at 10 and 100 s^−1^ and the flow behavior index were used for comparison.

For temperature-response tests, DHP-M particles were hydrated in 100,000 mg/L simulated formation water at 60, 80, 100, and 120 °C for 24 h. For salinity-response tests, DHP-M particles were hydrated in simulated formation waters with total salinities of 50,000, 100,000, and 150,000 mg/L at 60 °C for 24 h. The same 100,000 mg/L simulated formation water was used for core saturation, baseline water flooding, hydrogel particle dispersion, and chase-water flooding unless otherwise stated.

### 4.5. Structural and Morphological Characterization

#### 4.5.1. FTIR Characterization

Fourier transform infrared spectroscopy (FTIR) was used to identify the main functional groups of the deformable hydrogel particles and to assess whether the resulting spectra were consistent with the expected AM/AMPS/AA-based network chemistry. Before analysis, DHP-S, DHP-M, DHP-L, DHP-M-LC, and DHP-M-HC were dried at 45 °C for 24 h and gently ground into fine powders. The powders were directly placed on the attenuated total reflection accessory without KBr dilution. FTIR spectra were collected over the range of 4000–500 cm^−1^ at a resolution of 4 cm^−1^, with 32 scans accumulated for each sample. A background spectrum was recorded before each measurement.

#### 4.5.2. SEM Morphological Observation

The particle morphology and internal porous structure were observed using scanning electron microscopy (SEM). For overall particle morphology, hydrated particles were separated from the dispersion using a 100-mesh sieve, gently rinsed with deionized water, and dried under vacuum at 45 °C. For internal network observation, swollen particles were rapidly frozen in liquid nitrogen, fractured, and freeze-dried for 24 h. The dried samples were fixed on conductive adhesive tape and sputter-coated with gold before observation. SEM images were acquired at an accelerating voltage of 5 kV. The average pore diameter of freeze-dried particles was calculated from at least three representative regions using ImageJ software (version 1.54p; National Institutes of Health, Bethesda, MD, USA). Because the SEM procedure required freezing, freeze-drying, and vacuum imaging, the resulting pore-size values were used only as comparative descriptors of the freeze-dried network morphology and were not interpreted as direct measurements of the internal pore dimensions of fully hydrated particles.

For core-flooding samples, representative fragments were collected from the inlet, middle, and outlet sections of selected cores after transport experiments. The fragments were dried at 45 °C and mounted to expose internal pore-throat surfaces. Retained particles, pore-throat constrictions, and local accumulation features were observed under the same SEM conditions.

#### 4.5.3. Particle Size Distribution Measurement

The particle size distributions of dry and hydrated hydrogel particles were measured using laser particle size analysis and checked by optical microscopy. For dry particles, approximately 0.05 g of each sample was dispersed in anhydrous ethanol and stirred gently for 5 min. Strong ultrasonication was not applied to avoid breaking weakly crosslinked particles or altering the original particle size distribution.

For hydrated particles, 0.10 g of dry sample was dispersed in 100 mL of simulated formation water and hydrated at 60 °C for 24 h. After hydration, the particle suspension was directly transferred into the measurement cell, and the same simulated formation water was used as the dispersing medium. D10, D50, and D90 were recorded for each sample. D50 was used as the characteristic particle size for pore-throat matching analysis, while D10 and D90 were used to describe the distribution width. Particle size measurements were performed using three independently synthesized batches for each formulation (*n* = 3); repeated instrumental readings within each batch were averaged as technical replicates.

### 4.6. Swelling and Deformation Recovery Tests

#### 4.6.1. Swelling Test

The swelling behavior of hydrogel particles was evaluated by the gravimetric method. In each test, 0.10 g of dried hydrogel particles was dispersed in 100 mL of simulated formation water. For swelling kinetics, the hydration times were set as 0.5, 1, 2, 4, 8, 12, and 24 h at 60 °C. To evaluate temperature response, DHP-M particles were hydrated at 60, 80, 100, and 120 °C for 24 h. To evaluate salinity response, DHP-M particles were hydrated in simulated formation waters with total salinities of 50,000, 100,000, and 150,000 mg/L at 60 °C for 24 h. These 24 h tests were designed to compare equilibrium swelling and short-term environmental response among the tested conditions; they were not intended to represent long-term reservoir-aging tests.

At each scheduled time, the hydrated particles were separated using a 100-mesh sieve. Surface water was carefully removed with filter paper without pressing the swollen particles, and the particles were weighed immediately. The mass swelling ratio was calculated using Equation (1):

(1) $$S_{m}=\frac{m_{t}-m_{0}}{m_{0}}$$

where *S*_m_ is the mass swelling ratio, *m*_0_ is the initial dry particle mass, and *m*_t_ is the particle mass after hydration for time *t*.

The particle size swelling ratio was calculated from the change in D50 using Equation (2):

(2) $$S_{D}=\frac{D_{50,t}-D_{50,0}}{D_{50,0}}$$

where *S*_D_ is the particle size swelling ratio, *D*_50,0_ is the median particle size before hydration, and *D*_50,t_ is the median particle size after hydration. Swelling tests were performed independently using samples from three separate synthesis batches for each formulation (*n* = 3).

#### 4.6.2. Compression and Elastic Recovery Test

The compression and elastic recovery behavior of hydrated particles was measured using a universal testing machine. Dried hydrogel particles were hydrated in simulated formation water at 60 °C for 24 h. After hydration, the particles were separated from excess brine, and free surface water was gently removed with filter paper without mechanically squeezing the particles. The swollen particles were then transferred into a cylindrical mold with an inner diameter of 25 mm and lightly packed to form a hydrated particle bed with an initial height of approximately 20 mm. The particle bed was leveled without deliberate compaction, and the upper platen was brought into gentle contact with the sample surface before the initial bed height, *h*_0_, was recorded.

Compression was performed at a loading rate of 1 mm/min. The compression force and platen displacement were recorded continuously during loading. Compressive stress was calculated from the measured force divided by the cross-sectional area of the 25 mm-diameter particle bed. Engineering compressive strain was calculated relative to the initial bed height. The particle bed was compressed to 50% strain, corresponding to a compressed height *h*_c_ = 0.5*h*_0_, and held at this deformation for 60 s. The specimen was then unloaded at the same crosshead rate of 1 mm/min until the applied compressive force returned to zero. After complete unloading, the particle bed was allowed to recover freely for 10 min without external load, after which the recovered height, *h*_r_, was recorded. The elastic recovery ratio was calculated using Equation (3):

(3) $$R_{e}=\frac{h_{r}-h_{c}}{h_{0}-h_{c}}\times 100\%$$

where *R*_e_ is the elastic recovery ratio, *h*_0_ is the initial height of the hydrated particle bed, *h*_c_ is the height at 50% compression, and *h*_r_ is the recovered height after unloading. Accordingly, *R*_e_ = 0% corresponds to no height recovery after unloading, whereas *R*_e_ = 100% corresponds to complete recovery to the initial bed height. Compression and elastic-recovery tests were performed on independently prepared specimens from three separate synthesis batches for each formulation (*n* = 3).

To evaluate the retention of recoverable deformability under repeated mechanical loading, DHP-M was further subjected to ten consecutive compression–unloading cycles using the same specimen preparation and geometry. Each hydrated particle bed was compressed to 50% engineering strain at 1 mm/min, held for 60 s, and unloaded at 1 mm/min until the compressive force returned to zero. A zero-load recovery period of 60 s was allowed between consecutive cycles. The compressive stress at 50% strain and the recovered bed height were recorded for each cycle. The cycle-specific elastic recovery ratio was calculated as follows:

(4) $$R_{e,i}=\frac{h_{r,i}-h_{c,i}}{h_{0,i}-h_{c,i}}\times 100\%$$

and the compressive stress at 50% strain was calculated as follows:

(5) $$\sigma_{50,i}=\frac{F_{50,i}}{A},\;A=\frac{\pi D^{2}}{4}$$

where *h*_0,i_, *h*_c,i_, and *h*_r,i_ are the particle-bed heights before compression, at 50% compression, and after 60 s of zero-load recovery in cycle *i*, respectively; *F*_50,i_ is the compressive force at 50% strain; and *D* is the mold diameter of 25 mm. After the tenth cycle, the specimen was allowed to recover for an additional 10 min before the final recovered height was recorded. Three independently synthesized DHP-M batches were tested (*n* = 3).

### 4.7. Pore-Throat Structure Characterization of Tight Cores

Natural tight sandstone cores from the Ordos Basin were used for pore-throat characterization and subsequent flow experiments. The cores were cut into cylindrical plugs with a diameter of 25 mm and a length of 50 mm. For each individual core plug, the actual diameter and length were measured before testing, and the corresponding pore volume was calculated from the measured geometric bulk volume and porosity. Mineralogical composition was determined from powdered material collected from the end-trim portion corresponding to each core plug by X-ray diffraction, with quartz, feldspar, clay minerals, and carbonate minerals used as the principal mineral groups. The dimensions, porosity, water permeability, pore volume, and mineralogical composition of every core used in the pore-throat characterization, single-core transport, heterogeneous flow diversion, and displacement experiments are provided in Supplementary Table S1. Before testing, the cores were cleaned, dried at 60 °C to constant mass, and vacuum-saturated with simulated formation water. Porosity was calculated from the dry and saturated masses, and water permeability was measured by steady-state water flooding.

Pore-throat characteristics were determined using mercury intrusion porosimetry, low-field nuclear magnetic resonance (NMR), and micro-computed tomography (micro-CT). Mercury intrusion tests were conducted on small core fragments to obtain throat-size distributions and median throat radii. The characteristic throat diameter was calculated as twice the median throat radius. Based on water permeability and characteristic throat diameter, the cores were classified into OTS-L, OTS-M, and OTS-H groups, representing progressively increased pore-throat confinement.

NMR measurements were performed on fully saturated core plugs using the MacroMR12-150H-I low-field NMR analyzer to obtain the saturated-state T_2_ distributions. After the initial NMR measurement, the saturated cores were centrifuged at an equivalent centrifugal pressure of 2.87 MPa (417 psi) for 1.5 h at 25 °C to remove movable water while retaining capillary-bound water. The cores were then immediately remeasured under the same NMR acquisition conditions to obtain the post-centrifugation T_2_ spectra. The T_2_ cutoff was determined from the cumulative signal distributions before and after centrifugation. Specifically, T_2c_ was defined as the relaxation time at which the cumulative signal amplitude of the fully saturated spectrum equaled the total remaining NMR signal after centrifugation:

(6) $$\sum_{T_{2,i}\leq T_{2c}}A_{\mathrm{sat},i}=\sum_{i}A_{\mathrm{cen},i}$$

where *A*_sat,i_ is the NMR signal amplitude of the fully saturated core at the ith T_2_ interval, *A*_cen,i_ is the corresponding signal amplitude after centrifugation, and T_2c_ is the cutoff relaxation time. Signals at T_2_ ≤ T_2c_ were assigned to bound fluid, whereas signals at T_2_ > T_2c_ were assigned to movable fluid. The T_2_ cutoff was determined individually for each core, and the group-averaged values are reported in Table 5. No direct conversion of T_2_ relaxation time to absolute pore radius was performed because surface relaxivity was not independently calibrated for these cores; therefore, T_2_ distributions were used primarily to compare relative fluid confinement and mobility among samples.

Selected cores were further scanned by micro-CT to reconstruct three-dimensional pore networks and identify connected pore-throat pathways.

### 4.8. Deformation-Informed Pore-Throat Confinement Analysis

To quantify the pore-throat matching relationship between deformable hydrogel particles and Ordos tight cores, two matching coefficients were introduced by considering both hydrated particle size and particle deformability. The characteristic throat diameter of the core, d_throat, was calculated as twice the median throat radius obtained from mercury intrusion porosimetry. Hydrated particle size was represented by the median diameter *D*_50,h_, while *D*_10,h_ and *D*_90,h_ were used to describe the distribution width of the hydrated particles.

First, a static particle/throat matching coefficient, λ, was defined as follows:

(7) $$\lambda =\frac{D_{50,h}}{d_{\mathrm{throat}}}$$

where *D*_50,h_ is the hydrated median particle diameter of the hydrogel particles, and *d*_throat_ is the characteristic throat diameter of the tight core. A higher λ value indicates a larger size mismatch between hydrated particles and pore throats under the assumption of rigid-particle passage. To account for particle size distribution breadth within the same framework, lower- and upper-distribution apparent confinement descriptors, *λ*_app,10_ and *λ*_app,90_, were additionally calculated by substituting D_10,h_ and D_90,h_, respectively, for D_50,h_ in the *λ*_app_ expression. These values were used to define the confinement envelope associated with the measured particle size distribution rather than as independent regime thresholds.

Because the prepared hydrogel particles are deformable rather than rigid grains, bulk compression was used to provide a standardized experimental descriptor of the mechanical response of the hydrated particle assembly. However, the measured bed strain cannot be equated directly with the deformation of an individual hydrogel particle because it contains contributions from particle deformation, interparticle rearrangement, void closure, and fluid redistribution. Therefore, rather than defining a physically contracted single-particle diameter, a bulk strain-adjusted apparent size proxy, *D*_app_, was introduced solely for comparative normalization. For a one-dimensional engineering strain, the dimension along the loading direction follows the kinematic relation *L* = *L*_0_(1 − *ε*_b_); this relation was therefore used as a first-order normalization:

(8) $$D_{\mathrm{app}}=D_{50,h}\times (1-\varepsilon_{b})$$

where *ε*_b_ is the prescribed bulk strain of the hydrated particle bed. In the present study, *ε*_b_ = 0.50 corresponds to the standardized 50% compression condition used in the mechanical tests. Importantly, *D*_app_ should not be interpreted as the experimentally measured minimum transverse diameter of an individual particle during pore-throat extrusion. Particle-scale extrusion involves finite deformation, particle–wall contact, interfacial friction, local dehydration, and pressure-dependent shape evolution, which cannot be resolved by the present bulk-compression experiment [34,38].

Based on *D*_app_, a bulk strain-adjusted apparent confinement coefficient, *λ*_app_, was calculated as:

(9) $$\lambda_{app}=\frac{D_{app}}{d_{\mathrm{throat}}}=\frac{D_{50,h}\times (1-\varepsilon_{b})}{d_{\mathrm{throat}}}$$

Because the same standardized bulk strain (*ε*_b_ = 0.50) was applied to DHP-S, DHP-M, and DHP-L, *λ*_app_ = 0.5λ for all three particle size groups. Thus, this normalization changes the magnitude of the size-mismatch descriptor but does not alter the relative ordering among the hydrogel systems. *λ*_app_ was therefore used only to compare relative confinement under an identical bulk-compression reference state, rather than to predict whether an individual particle can geometrically pass through a specific throat. The *λ*_app_ value was interpreted together with the hydrated particle size distribution, throat-size distribution, compressive response, elastic recovery, effluent particle recovery, and axial retention profile. When *λ*_app_ ≫ 1, free passage through the median throat is physically unlikely; experimentally observed transport is instead interpreted as selective propagation through the accessible and relatively larger connected portion of the pore network, accompanied by deformation-mediated trapping, release, and re-migration.

The uncertainty of the derived matching coefficients was evaluated by first-order error propagation, assuming that the experimental variations in hydrated particle size and characteristic throat diameter were independent. The standard deviations of *D*_50,h_ and *d*_throat_ obtained from the independent measurements were used as the corresponding uncertainty terms. For the static matching coefficient, the propagated uncertainty was calculated as:

(10) $$u_{\lambda }=\lambda \sqrt{{\left(\frac{u_{D}}{D_{50,h}}\right)}^{2}+{\left(\frac{u_{d}}{d_{\mathrm{throat}}}\right)}^{2}}$$

where *u*_D_ and *u*_d_ are the standard deviations of the hydrated median particle diameter and characteristic throat diameter, respectively. Because *ε*_b_ = 0.50 was a prescribed bulk-compression reference condition rather than an independently measured random variable, no additional statistical uncertainty was assigned to this fixed normalization term. Under the present definition, *λ*_app_ = 0.5*λ*, and therefore:

(11) $$u_{\lambda_{\mathrm{app}}}=0.5u_{\lambda }$$

Accordingly, *λ* and *λ*_app_ have the same relative propagated uncertainty, whereas the absolute uncertainty of *λ*_app_ is one-half that of *λ*. The propagated uncertainty was used to evaluate the robustness of the relative confinement ranking rather than to redefine the empirical regime boundaries.

Because the 50% bulk strain was used as a standardized reference condition rather than as a measured single-particle deformation, the sensitivity of *λ*_app_ to the selected reference strain was additionally evaluated. Representative bulk strain levels of 20%, 30%, 40%, and 50%, all within the experimentally applied compression range, were considered using *D*_app_ = *D*_50,h_(1 − *ε*_b_). The resulting λapp values were compared to assess whether the relative confinement ranking and qualitative regime interpretation depended strongly on the selected normalization strain. This analysis was used only as a sensitivity test and does not imply that individual hydrogel particles experience these exact deformation levels during pore-throat passage.

For construction of the regime map in Figure 6d, two empirical operational boundaries were defined at *λ*_app_ = 25 and *λ*_app_ = 80. These boundaries were selected from the separation between the low-, intermediate-, and high-confinement groups formed by the tested hydrogel–core combinations and were subsequently evaluated against the independently measured pressure response, particle-retention distribution, and effluent particle recovery. Thus, *λ*_app_ < 25, 25 ≤ *λ*_app_ < 80, and *λ*_app_ ≥ 80 were used to denote migration-dominated transport with weak retention, adaptive migration with selective retention, and high confinement with premature-plugging risk, respectively. These boundaries are specific to the particle formulations, tight core structures, fluid conditions, and injection protocol employed in this study and should not be extrapolated as universal critical matching coefficients.

### 4.9. Injectability and Transport Test in Tight Cores

Injectability and transport behavior were evaluated with a high-temperature high-pressure core-flooding system. Each core was dried, vacuum-saturated with simulated formation water, and mounted in the core holder. The confining pressure was kept at least 5 MPa higher than the inlet pressure to avoid bypass flow. Unless otherwise specified, the test temperature was 60 °C, and the injection rate was 0.20 mL/min.

Simulated formation water was first injected until the pressure drop across the core reached a stable value. This stage was used to determine the baseline permeability. A 0.30 wt% hydrogel particle dispersion was then injected into the core. The target injection volume was 0.30 pore volume (PV), and the actual injected dispersion volume and corresponding dry hydrogel mass were recorded for each individual core. All single-core flooding experiments reported in this study completed the prescribed 0.30 PV injection; therefore, no reported test required early termination because of excessive pressure buildup. The core-specific pore volume, actual injected dispersion volume, and dry hydrogel mass are summarized in Supplementary Table S2. After particle injection, simulated formation water was injected again to evaluate the residual flow resistance caused by particle retention. Inlet pressure, outlet pressure, injection volume, and effluent volume were recorded throughout the experiment.

The resistance factor and residual resistance factor were calculated using Equations (7) and (8):

(12) $$RF=\frac{\Delta P_{g}}{\Delta P_{w}}$$

(13) $$RRF=\frac{\Delta P_{w,after}}{\Delta P_{w,before}}$$

where *RF* is the resistance factor during hydrogel particle injection, Δ*P*_g_ is the pressure drop during particle injection, Δ*P*_w_ is the pressure drop during baseline water flooding, RRF is the residual resistance factor, Δ*P*_w,before_ is the pressure drop before hydrogel treatment, and Δ*P*_w,after_ is the pressure drop during subsequent water flooding after hydrogel treatment.

Particle breakthrough and retention were quantified by dry-particle mass balance. Before each core-flooding experiment, the actual dry-particle concentration of the injected hydrogel dispersion was verified gravimetrically using a known-volume aliquot. The aliquot was vacuum-filtered through a pre-weighed 0.45 μm membrane, rinsed thoroughly with deionized water to remove soluble salts, and dried at 45 °C to constant mass. The total injected dry-particle mass, m_in, was determined from the measured particle concentration and the actual injected dispersion volume.

Effluent samples were collected continuously in separate fractions during hydrogel injection and subsequent water flooding. Each fraction was processed using the same filtration, deionized-water rinsing, and constant-mass drying procedure. The dry hydrogel mass recovered from all effluent fractions was summed to obtain the cumulative produced-particle mass, m_out. The whole-core particle-retention efficiency was calculated as:

(14) $$\eta_{\mathrm{ret}}=\frac{m_{\mathrm{in}}-m_{\mathrm{out}}}{m_{\mathrm{in}}}\times 100\%$$

and the cumulative particle-recovery ratio was calculated as:

(15) $$R_{p}=\frac{m_{\mathrm{out}}}{m_{\mathrm{in}}}\times 100\%$$

where *η*_ret_ is the whole-core particle-retention efficiency and *R*_p_ is the cumulative fraction of injected dry hydrogel recovered from the effluent.

For axial retention analysis, post-flood cores were sectioned into five equal 10 mm-long segments from the inlet to the outlet, corresponding to normalized axial positions x/L of 0.1, 0.3, 0.5, 0.7, and 0.9. The dry mass of each core segment was recorded. Retained hydrogel particles were recovered from each segment by repeated aqueous extraction under gentle agitation. After mineral fragments had settled, the particle-containing supernatant was collected and vacuum-filtered through pre-weighed membranes. Extraction was repeated three times, and the collected membranes were rinsed thoroughly with deionized water to remove soluble salts and dried at 45 °C to constant mass. Untreated core fragments were processed using the same procedure for blank correction of mineral fines. The local retention density was calculated as:

(16) $$q_{i}=\frac{m_{\mathrm{ret},i}}{m_{\mathrm{core},i}}$$

where *q*_i_ is the dry hydrogel retention density in segment *i* (mg/g core), *m*_ret,i_ is the blank-corrected dry hydrogel mass recovered from the corresponding segment (mg), and *m*_core,i_ is the dry mass of that core segment (g). The sum of the segment-wise retained hydrogel masses was compared with the whole-core mass-balance value, *m*_in_ − *m*_out_, as an internal consistency check.

After the quantitative retention measurements, representative fragments from the inlet, middle, and outlet regions of selected cores were examined by SEM, optical microscopy, or micro-CT to visualize retained particles, local pore-throat constrictions, and accumulation patterns. These imaging methods were used as complementary spatial evidence rather than as the quantitative basis for the reported particle-retention efficiency or mg/g retention density.

To directly compare the core-scale transport behavior of the three particle size fractions, additional comparative flooding experiments were conducted using matched OTS-M cores. DHP-S, DHP-M, and DHP-L were evaluated separately under identical conditions. For each hydrogel system, three OTS-M core plugs with comparable porosity, permeability, and pore-throat characteristics were used. The hydrogel concentration, simulated formation-water composition, temperature, injection rate, and particle-injection volume were maintained at 0.30 wt%, 100,000 mg/L, 60 °C, 0.20 mL/min, and 0.30 PV, respectively. Baseline water flooding, hydrogel injection, and subsequent water flooding were performed following the same procedure described above. Injection-pressure response, resistance factor, residual resistance factor, particle-retention efficiency, cumulative effluent particle recovery, and axial retention distribution were compared among DHP-S, DHP-M, and DHP-L. A focused rate-sensitivity series was additionally conducted using DHP-M and matched OTS-M cores at injection rates of 0.10, 0.20, and 0.30 mL/min, while the hydrogel concentration, brine salinity, temperature, and particle-injection volume were maintained at 0.30 wt%, 100,000 mg/L, 60 °C, and 0.30 PV, respectively (*n* = 3 per rate).

### 4.10. Flow-Redistribution Test in Heterogeneous Parallel Core Systems

Flow-redistribution behavior under controlled permeability contrast was investigated using a parallel core flooding system. Two tight sandstone cores with different permeabilities were connected in parallel to simulate heterogeneous flow channels. Before testing, both cores were vacuum-saturated with simulated formation water and mounted in the parallel core holder. The permeability contrast between the high-permeability and low-permeability cores was controlled within a preset range to represent the heterogeneity of tight reservoirs.

Simulated formation water was injected first at a total flow rate of 0.30 mL/min until stable flow was obtained. Effluents from the high-permeability and low-permeability cores were collected separately to determine the initial flow distribution. A 0.30 wt% DHP-M particle dispersion corresponding to 0.30 PV of the total connected pore volume of the parallel core assembly was then injected into the parallel system, followed by subsequent water flooding. The actual dispersion volume and corresponding dry DHP-M mass for each parallel core assembly are reported in Supplementary Table S2. No parallel core experiment required premature termination. During the test, the pressure response and effluent volume from each core were recorded.

The flow fraction of each core was calculated using Equation (9):

(17) $$F_{j}=\frac{Q_{j}}{Q_{H}+Q_{L}}\times 100\%$$

where *F*_j_ is the flow fraction of the high-permeability or low-permeability core, *Q*_j_ is the effluent flow rate from the corresponding core, *Q*_H_ is the effluent flow rate from the high-permeability core, and *Q*_L_ is the effluent flow rate from the low-permeability core. The reduction in flow fraction through the high-permeability core and the increase in flow fraction through the low-permeability core were used to evaluate the flow diversion capacity of the hydrogel particles.

After the parallel core experiments, the high-flow and low-flow cores were processed separately using the same gravimetric hydrogel-recovery procedure described in Section 4.9. The dry hydrogel mass retained in each branch was normalized by the dry mass of the corresponding core to obtain the branch-specific retention density in (mg/g core).

### 4.11. Displacement and Movable Fluid Redistribution Test

Displacement experiments were conducted to evaluate whether DHP-M-assisted flow diversion could improve sweep efficiency and movable fluid utilization. The cores were first vacuum-saturated with simulated formation water and then displaced with a viscosity-matched model oil until no water was produced. At the experimental temperature of 60 °C, the model oil had a viscosity of 8.5 mPa·s and a density of 0.836 g/cm^3^; its interfacial tension against the 100,000 mg/L simulated formation water was 27.2 mN/m. The oil-saturated cores were aged at 60 °C for 24 h to stabilize the initial fluid distribution.

Primary water flooding was performed at 0.20 mL/min until the water cut reached 98% or oil production became negligible. Then, 0.30 wt% DHP-M particle dispersion was injected at the same rate, with an injection volume of 0.30 PV. The actual injected dispersion volume and dry DHP-M mass for each displacement experiment are provided in Supplementary Table S2. After particle injection, the cores were shut in for 12 h to allow particle retention and local flow resistance development. Subsequent water flooding was conducted until both pressure and produced liquid volume became stable. Oil and water volumes were recorded at each stage. Incremental oil recovery was defined as the absolute increase in the OOIP-based recovery factor from the end of primary water flooding to the end of subsequent water flooding after DHP-M treatment and is therefore reported in percentage points rather than as a relative percentage increase.

Untreated parallel core controls were conducted to distinguish the contribution of DHP-M from continued water flooding and fluid redistribution during shut-in. For each HCS-LM, HCS-LH, and HCS-MH configuration, three independently assembled matched parallel core systems were subjected to the same saturation, aging, and primary water-flooding procedures as the DHP-M-treated systems. After primary water flooding reached the same termination criterion, 0.30 PV of simulated formation water without hydrogel particles was injected at 0.20 mL/min in place of the 0.30 wt% DHP-M dispersion. The control systems were then shut in for 12 h and subjected to subsequent water flooding under the same injection conditions and termination criteria as the DHP-M-treated systems. Branch flow fraction, movable fluid saturation, and cumulative oil recovery were measured at the corresponding experimental stages. These brine-only controls were used to quantify changes attributable to continued water flooding and capillary redistribution in the absence of hydrogel treatment.

Low-field NMR measurements were performed before water flooding, after primary water flooding, and after subsequent water flooding following DHP-M treatment. The T_2_ spectra were analyzed in short-, intermediate-, and long-relaxation intervals. Because T_2_ relaxation in water-saturated sandstone is influenced not only by pore size but also by surface relaxivity, pore geometry, and fluid–solid interactions, these intervals were not directly converted into absolute pore-size classes. Under the same lithology and saturation-fluid conditions, shorter T_2_ components were interpreted qualitatively as representing more strongly confined fluid domains, whereas longer T_2_ components were associated with relatively weakly confined and more accessible pore domains. Changes in signal intensity within these intervals were therefore used to evaluate relative fluid redistribution rather than to assign quantitative pore sizes. Changes in signal intensity across these intervals were used to evaluate movable fluid redistribution after hydrogel-induced flow diversion.

### 4.12. Microscopic Visualization of Seepage Pathway Reconstruction

Micro-CT was used to comparatively visualize the three-dimensional connected pore structure before and after hydrogel treatment. Selected cores were scanned using a SkyScan 1272 micro-computed tomography system (Bruker, Kontich, Belgium) at 100 kV and 100 μA with a 0.11 mm Cu filter. Projections were acquired over 360° with a rotation step of 0.2° and a frame averaging of 3, yielding an isotropic voxel size of 8.0 μm. For comparative analysis, an 8.0 × 8.0 × 8.0 mm^3^ central volume of interest, corresponding to 1000 × 1000 × 1000 voxels, was extracted from each reconstructed dataset while avoiding the external core boundary. Before- and after-treatment datasets from the same core were spatially registered to the same volume of interest. Reconstruction was performed using NRecon software (version 1.7.4.2; Bruker microCT, Kontich, Belgium) with identical settings for all datasets, including a smoothing level of 2, ring-artifact correction of 8, and beam-hardening correction of 20%. Reconstructed grayscale images were processed using CTAn software (version 1.20.3.0; Bruker microCT, Kontich, Belgium), and the pore phase was separated from the solid matrix using the IsoData automatic thresholding method followed by removal of isolated single-voxel noise. The same segmentation procedure was applied to the corresponding pre- and post-treatment datasets. Connected porosity was calculated as the volume fraction of the segmented pore phase belonging to the inlet-to-outlet percolating cluster within the registered volume of interest. Geometric tortuosity was calculated as the ratio of the shortest connected pore-path length between the inlet and outlet faces to the corresponding straight-line distance. The same registered volume of interest and segmentation procedure were used for the pre- and post-treatment datasets. Because the voxel size imposes a finite spatial-resolution limit, the uncertainty in linear feature dimensions was conservatively taken as ±1 voxel (±8 μm), and features smaller than two voxels (~16 μm) were not interpreted quantitatively. Accordingly, the micro-CT results were used primarily to compare the continuity and spatial organization of resolvable connected pore pathways rather than to quantify submicron pore throats. After core flooding, representative fragments from the inlet, middle, and outlet sections were collected for SEM observation. The fragments were dried at 45 °C and sectioned carefully to expose the internal pore-throat surfaces. Special attention was given to particle retention at pore-throat constrictions, particle accumulation in relatively large channels, and changes in local pore connectivity. These observations were combined with pressure-response and NMR results to interpret the pore-throat-scale process of deformable migration, selective retention, and flow diversion.

### 4.13. Data Processing and Statistical Analysis

Unless otherwise stated, *n* = 3 refers to three independent experimental replicates rather than repeated instrumental readings of the same specimen. For hydrogel characterization, each formulation was prepared in three independent synthesis batches. Particle size, swelling, and compression/recovery results were obtained from independently prepared specimens from these three batches. Where repeated instrumental readings were required within one specimen, these readings were treated as technical replicates and averaged before statistical analysis and were not counted as independent values of *n*. For SEM pore-size quantification, multiple image fields from different particles were analyzed within each synthesis batch and averaged to obtain one batch-level value; therefore, *n* = 3 represents three independently synthesized batches rather than three SEM fields.

For pore-throat characterization and single-core flooding experiments, *n* = 3 represents three separate core plugs for each core group or hydrogel–core condition. For heterogeneous parallel core flow diversion and displacement experiments, *n* = 3 represents three independently assembled parallel core systems using separate core plugs. Matching and confinement coefficients calculated from the hydrogel and core parameters are derived quantities and do not constitute additional independent replicates. Results are reported as mean ± standard deviation, and error bars represent the standard deviation among the corresponding independent experimental replicates.

For particle size distribution, D10, D50, D90, and span values were used to describe particle size characteristics, and the span value was calculated using Equation (10):

(18) $$Span=\frac{D_{90}-D_{10}}{D_{50}}$$

Core permeability was calculated according to Darcy’s law using Equation (11):

(19) $$k=\frac{Q\mu L}{A\Delta P}$$

where *k* is the permeability, *Q* is the flow rate, *μ* is the fluid viscosity, *L* is the core length, *A* is the cross-sectional area, and Δ*P* is the pressure drop across the core.

Particle size images, pore-throat morphology, and retained-particle distributions were analyzed using ImageJ. NMR T_2_ spectra were processed by dividing the relaxation signals into pore-size-related intervals, and the signal proportion in each interval was calculated from repeated measurements. For core-flooding and parallel core experiments, pressure profiles are presented as representative individual runs selected from three independent experiments for each condition rather than as averaged curves. All summarized quantitative parameters derived from independent replicates are reported as mean ± standard deviation, and the corresponding figure error bars represent standard deviations. Statistical differences among three or more independent groups were evaluated using one-way analysis of variance (one-way analysis of variance (ANOVA)). When the overall ANOVA indicated a significant group effect, Tukey’s honestly significant difference (HSD) test was subsequently applied for pairwise comparisons. Paired before–after measurements obtained from the same core or parallel core system were evaluated using two-tailed paired *t*-tests. Exact *p*-values, together with the corresponding ANOVA F statistics and degrees of freedom where applicable, are reported for the principal statistical comparisons in the Results and Discussion Section. Two-sided 95% confidence intervals were calculated for the corresponding mean differences. Statistical significance was defined as *p* < 0.05. Where significance indicators are shown in the figures, different lowercase letters denote significant differences among multiple groups according to Tukey’s HSD test, whereas asterisks indicate significant paired before–after differences.

## Figures and Tables

**Table 1:** Main FTIR absorption bands of deformable hydrogel particles and their assignments.

Wavenumber/cm^−1^	Assignment	Structural Origin	Functional Implication
3430–3442	–NH/–OH stretching	AM, AMPS, and AA units	Hydration sites and water affinity
1710–1722	C=O stretching of –COOH/–COO^−^	AA units	Swelling regulation and ionizable groups
1652–1658	Amide I, C=O stretching	AM, AMPS, and MBAA units	Acrylamide-based network structure
1542–1548	Amide II, N–H bending	AM, AMPS, and MBAA units	Amide-containing copolymer network
1186–1195	Asymmetric S=O stretching	AMPS units	Salt-tolerant sulfonic groups
1038–1048	Symmetric S=O stretching	AMPS units	Hydrophilic ionic groups

**Table 2:** Pore-structure parameters of DHP-M samples with different crosslinking densities obtained from SEM image analysis.

Sample	MBAA Dosage/wt% of Monomers	Average Pore Diameter/μm	Morphological Feature	Structural Implication
DHP-M-LC	0.20	18.7 ± 2.3	Loose network with larger pores	Higher deformation potential
DHP-M	0.30	12.9 ± 1.6	Moderately crosslinked porous network	Balanced network structure
DHP-M-HC	0.50	7.6 ± 1.1	Dense network with smaller pores	Higher rigidity and lower deformation potential

**Table 3:** Dry and hydrated particle size parameters of deformable hydrogel particles.

Sample	Emulsification Speed/rpm	MBAA Dosage/wt% of Monomers	Dry D10/μm	Dry D50/μm	Dry D90/μm	Hydrated D10/μm	Hydrated D50/μm	Hydrated D90/μm	Hydrated Span
DHP-S	12,000	0.30	17.8 ± 1.3	34.6 ± 2.1	65.2 ± 4.4	28.5 ± 2.0	58.3 ± 3.5	106.1 ± 6.8	1.33
DHP-M	8000	0.30	39.6 ± 2.7	72.8 ± 4.6	126.5 ± 7.9	61.7 ± 3.9	118.6 ± 6.9	202.8 ± 11.7	1.19
DHP-L	5000	0.30	78.4 ± 5.1	138.5 ± 8.7	234.8 ± 13.6	121.4 ± 7.2	224.7 ± 12.5	370.6 ± 19.8	1.11
DHP-M-LC	8000	0.20	40.3 ± 2.9	74.1 ± 4.8	129.6 ± 8.4	72.5 ± 4.6	137.4 ± 8.2	229.6 ± 13.3	1.14
DHP-M-HC	8000	0.50	38.9 ± 2.5	71.6 ± 4.2	123.8 ± 7.5	52.1 ± 3.3	96.2 ± 5.7	160.4 ± 9.6	1.13

**Table 4:** Swelling and deformation recovery parameters of deformable hydrogel particles.

Sample	MBAA Dosage/wt% of Monomers	Equilibrium Swelling Ratio at 60 °C	Hydrated D50/μm	Compressive Stress at 50% Strain/kPa	Elastic Recovery Ratio/%
DHP-S	0.30	4.38 ± 0.29	58.3 ± 3.5	12.6 ± 0.9	80.4 ± 3.1
DHP-M	0.30	4.63 ± 0.31	118.6 ± 6.9	14.8 ± 1.1	81.6 ± 3.2
DHP-L	0.30	4.86 ± 0.34	224.7 ± 12.5	15.5 ± 1.2	79.2 ± 3.4
DHP-M-LC	0.20	5.76 ± 0.38	137.4 ± 8.2	9.7 ± 0.8	86.4 ± 3.5
DHP-M-HC	0.50	3.51 ± 0.24	96.2 ± 5.7	21.3 ± 1.5	70.8 ± 3.0

**Table 5:** Physical properties, pore-throat parameters, and movable fluid distribution of Ordos tight cores.

Core Group	Porosity/%	Water Permeability/mD	Median Throat Radius/μm	Characteristic Throat Diameter/μm	NMR T_2_ Cutoff/ms	Bound Fluid Saturation/%	Movable Fluid Saturation/%
OTS-L	11.64 ± 0.51	0.412 ± 0.046	0.62 ± 0.07	1.24 ± 0.14	10.0 ± 0.9	46.3 ± 2.6	53.7 ± 2.6
OTS-M	9.46 ± 0.44	0.216 ± 0.028	0.34 ± 0.04	0.68 ± 0.08	10.0 ± 1.0	63.8 ± 2.9	36.2 ± 2.9
OTS-H	7.82 ± 0.36	0.083 ± 0.012	0.18 ± 0.03	0.36 ± 0.06	10.0 ± 0.8	78.5 ± 3.1	21.5 ± 3.1

Note: Values in Table 5 represent group-level mean ± standard deviation (*n* = 3). Individual core dimensions, porosity, water permeability, pore volume, and mineralogical composition are provided in Supplementary Table S1.

**Table 6:** Key parameters used for pore-throat matching analysis between deformable hydrogels and Ordos tight cores.

Parameter	DHP-S	DHP-M	DHP-L	OTS-L	OTS-M	OTS-H
Hydrated D50/μm	58.3 ± 3.5	118.6 ± 6.9	224.7 ± 12.5	—	—	—
Compression strain used for correction	0.50	0.50	0.50	—	—	—
Bulk strain-adjusted apparent size, *D*_app_/μm	29.2	59.3	112.4	—	—	—
Elastic recovery ratio/%	80.4 ± 3.1	81.6 ± 3.2	79.2 ± 3.4	—	—	—
Characteristic throat diameter/μm	—	—	—	1.24 ± 0.14	0.68 ± 0.08	0.36 ± 0.06
Movable fluid saturation/%	—	—	—	53.7 ± 2.6	36.2 ± 2.9	21.5 ± 3.1
Bound fluid saturation/%	—	—	—	46.3 ± 2.6	63.8 ± 2.9	78.5 ± 3.1
Pore-network connectivity	—	—	—	High	Moderate	Low
Main matching implication	Easier entry but weaker retention	Balanced migration and retention	Stronger retention but higher plugging risk	Deep migration favored	Adaptive migration and retention favored	Strong confinement and plugging risk

Note: *D*_app_ was calculated as *D*_50,h_ × (1 − *ε*_b_), where *ε*_b_ = 0.50 corresponds to the standardized bulk strain applied to the hydrated particle bed. *D*_app_ is an apparent normalization parameter and does not represent the directly measured deformed diameter of an individual hydrogel particle. Characteristic throat diameter and movable fluid parameters were obtained from Section 2.3. Data are expressed as mean ± standard deviation where applicable, *n* = 3.

**Table 7:** Materials used in this study.

Material	Specification	Supplier	Role in System
Acrylamide (AM)	≥99.0%	Aladdin Biochemical Technology Co., Ltd., Shanghai, China	Main flexible monomer
2-Acrylamido-2-methylpropane sulfonic acid (AMPS)	≥98.0%	Macklin Biochemical Co., Ltd., Shanghai, China	Salt-tolerant functional monomer
Acrylic acid (AA)	≥99.0%	Aladdin Biochemical Technology Co., Ltd., Shanghai, China	Hydrophilic swelling-regulation monomer
N,N′-Methylenebisacrylamide (MBAA)	≥99.0%	Aladdin Biochemical Technology Co., Ltd., Shanghai, China	Crosslinking agent for network-density regulation
Ammonium persulfate (APS)	≥98.0%	Sinopharm Chemical Reagent Co., Ltd., Shanghai, China	Oxidizing initiator
Sodium bisulfite (NaHSO_3_)	≥98.0%	Xilong Scientific Co., Ltd., Shantou, China	Reducing initiator
Sodium hydroxide (NaOH)	≥96.0%	Xilong Scientific Co., Ltd., Shantou, China	pH adjustment
Liquid paraffin	Analytical grade	Sinopharm Chemical Reagent Co., Ltd., Shanghai, China	Continuous oil phase for inverse emulsion
Span 80	Chemical grade	Macklin Biochemical Co., Ltd., Shanghai, China	Lipophilic emulsifier
Tween 80	Chemical grade	Macklin Biochemical Co., Ltd., Shanghai, China	Hydrophilic emulsifier
Anhydrous ethanol	≥99.7%	Sinopharm Chemical Reagent Co., Ltd., Shanghai, China	Demulsification and washing medium
Petroleum ether	Boiling range: 60–90 °C	Xilong Scientific Co., Ltd., Shantou, China	Removal of residual oil phase
Sodium chloride (NaCl)	≥99.5%	Xilong Scientific Co., Ltd., Shantou, China	Simulated formation water component
Calcium chloride (CaCl_2_)	≥96.0%	Macklin Biochemical Co., Ltd., Shanghai, China	Simulated formation water component
Magnesium chloride hexahydrate (MgCl_2_·6H_2_O)	≥98.0%	Aladdin Biochemical Technology Co., Ltd., Shanghai, China	Simulated formation water component
Deionized water	Resistivity ≥ 18.2 MΩ·cm	Laboratory purification system	Solvent, washing medium, and aqueous phase
Tight sandstone cores	Ordos Basin; cylindrical cores	Provided by oilfield core repository	Pore-throat characterization and core flooding tests
Model oil	Viscosity-matched model oil	Provided by oilfield laboratory	Displacement and movable fluid redistribution tests

**Table 8:** Major instruments and testing systems.

Instrument	Model	Manufacturer	Application
Fourier transform infrared spectrometer	Nicolet iS50	Thermo Fisher Scientific, Waltham, MA, USA	Chemical structure analysis of hydrogel particles
Scanning electron microscope	JSM-IT500	JEOL Ltd., Tokyo, Japan	Morphological observation of dried hydrogel particles and pore-throat features
Optical microscope	DM2700 M	Leica Microsystems, Wetzlar, Germany	Hydrated particle morphology and image acquisition
Laser particle size analyzer	Mastersizer 3000	Malvern Panalytical, Malvern, UK	Particle size distribution measurement
Rotational rheometer	MCR 302	Anton Paar GmbH, Graz, Austria	Rheological characterization of hydrogel particle suspensions
Universal testing machine	CMT5105	MTS Industrial Systems Co., Ltd., Shenzhen, China	Compression and elastic recovery tests
Low-field nuclear magnetic resonance analyzer	MacroMR12-150H-I	Niumag Analytical Instrument Co., Ltd., Suzhou, China	Movable fluid distribution and T_2_ spectrum analysis
Micro-computed tomography system	SkyScan 1272	Bruker, Kontich, Belgium	Three-dimensional pore-throat structure and seepage-pathway visualization
Mercury intrusion porosimeter	AutoPore V 9600	Micromeritics Instrument Corp., Norcross, GA, USA	Pore-throat size distribution analysis
High-temperature drying oven	DHG-9070A	Shanghai Yiheng Scientific Instrument Co., Ltd., Shanghai, China	Sample drying and pretreatment
Thermostatic water bath	HH-6	Changzhou Guohua Electric Appliance Co., Ltd., Changzhou, China	Polymerization and hydration temperature control
High-speed homogenizer	T25 digital	IKA-Werke GmbH & Co. KG, Staufen, Germany	Emulsification and particle size regulation
High-temperature high-pressure core flooding system	HTHP-CF100	Jiangsu Hai’an Petroleum Scientific Instrument Co., Ltd., Hai’an, China	Injectability, transport, and retention tests in tight cores
Parallel core flow diversion system	PFM-3	Jiangsu Hai’an Petroleum Scientific Instrument Co., Ltd., Hai’an, China	Deep flow diversion tests in heterogeneous core systems
High-precision syringe pump	LSP01-1A	Baoding Longer Precision Pump Co., Ltd., Baoding, China	Controlled injection of hydrogel particle suspensions and fluids
Pressure transducer	CYB-20S	Beijing Star Sensor Technology Co., Ltd., Beijing, China	Real-time pressure monitoring during core flooding
Electronic balance	BSA224S	Sartorius, Göttingen, Germany	Mass measurement
pH meter	PHS-3C	Shanghai INESA Scientific Instrument Co., Ltd., Shanghai, China	pH adjustment and monitoring
Vacuum filtration device	SHZ-D(III)	Gongyi Yuhua Instrument Co., Ltd., Zhengzhou, China	Particle separation and washing
Vacuum drying oven	DZF-6050	Shanghai Yiheng Scientific Instrument Co., Ltd., Shanghai, China	Drying of hydrogel particles and core samples

**Table 9:** Formulation and design purpose of deformable hydrogel particles.

Sample	AM/g	AMPS/g	AA/g	MBAA/g	MBAA Dosage/wt% of Monomers	Emulsification Speed/rpm	Design Purpose
DHP-S	4.20	1.20	0.60	0.018	0.30	12,000	Small particles for deep migration
DHP-M	4.20	1.20	0.60	0.018	0.30	8000	Medium particles for balanced transport and retention
DHP-L	4.20	1.20	0.60	0.018	0.30	5000	Large particles for preferential-pathway retention
DHP-M-LC	4.20	1.20	0.60	0.012	0.20	8000	Low-crosslinking-density control for higher deformability
DHP-M-HC	4.20	1.20	0.60	0.030	0.50	8000	High-crosslinking-density control for lower deformability

## Data Availability

All data supporting the findings of this study are provided within the article and its Supplementary Materials. The underlying datasets for particle size distributions, core-flooding pressure responses, NMR T_2_ measurements, particle-retention analyses, and oil-recovery calculations are included in the Supplementary Data and will be publicly accessible together with the published article.

## Supplementary Materials

The following supporting information can be downloaded at: https://www.mdpi.com/article/10.3390/gels12100924/s1, Table S1. Physical and mineralogical properties of individual Ordos tight sandstone cores used in this study; Table S2. Actual hydrogel injection volumes and dry hydrogel masses used in the core-flooding experiments; Table S3. Comparison of DHP-M-treated and untreated control experiments in heterogeneous parallel-core systems; Table S4. Quantitative micro-CT parameters of representative Ordos tight sandstone cores before and after DHP-M treatment; Table S5. Effect of injection rate on DHP-M transport in matched OTS-M cores; Table S6. Rheological properties of 0.30 wt% DHP-M dispersions at 60 °C under different salinity conditions; Table S7. Sensitivity of the apparent confinement coefficient λapp to the prescribed bulk-strain reference level.

## Abbreviations

Abbreviation/Symbol	Definition
AA	Acrylic acid
AM	Acrylamide
AMPS	2-Acrylamido-2-methylpropane sulfonic acid
ANOVA	Analysis of variance
APS	Ammonium persulfate
DHP	Deformable hydrogel particle
DHP-S	Small-sized deformable hydrogel particles
DHP-M	Medium-sized deformable hydrogel particles
DHP-L	Large-sized deformable hydrogel particles
DHP-M-LC	Medium-sized deformable hydrogel particles with low crosslinking density
DHP-M-HC	Medium-sized deformable hydrogel particles with high crosslinking density
DPG	Dispersed particle gel
EGP	Elastic gel particle
FTIR	Fourier transform infrared spectroscopy
HCS	Heterogeneous core system
HCS-LM	Parallel heterogeneous system composed of OTS-L and OTS-M cores
HCS-LH	Parallel heterogeneous system composed of OTS-L and OTS-H cores
HCS-MH	Parallel heterogeneous system composed of OTS-M and OTS-H cores
HSD	Honestly significant difference
MBAA	N,N′-Methylenebisacrylamide
MIP	Mercury intrusion porosimetry
micro-CT	Micro-computed tomography
NMR	Nuclear magnetic resonance
OOIP	Original oil in place
OTS	Ordos tight sandstone
OTS-L	Ordos tight sandstone with low pore-throat confinement
OTS-M	Ordos tight sandstone with intermediate pore-throat confinement
OTS-H	Ordos tight sandstone with high pore-throat confinement
PPG	Preformed particle gel
PV	Pore volume
RF	Resistance factor
RRF	Residual resistance factor
SEM	Scanning electron microscopy
D10	Particle diameter below which 10% of the particle size distribution lies
D50	Median particle diameter
D90	Particle diameter below which 90% of the particle size distribution lies
*D*_50,h_	Hydrated median particle diameter
*D*_app_	Bulk strain-adjusted apparent particle size proxy
*d*_throat_	Characteristic throat diameter
*ε*_b_	Prescribed bulk-compression strain
*λ*	Static particle/throat matching coefficient
*λ*_app_	Bulk strain-adjusted apparent confinement coefficient
*λ*_app,10_	Apparent confinement coefficient calculated using hydrated D10
*λ*_app,90_	Apparent confinement coefficient calculated using hydrated D90
T_2_	Transverse relaxation time
T_2c_	NMR transverse-relaxation cutoff
*S*_m_	Mass swelling ratio
*S*_D_	Particle size swelling ratio
*R*_e_	Elastic recovery ratio
*η*_ret_	Whole-core particle-retention efficiency
*R*_p_	Cumulative particle-recovery ratio
*q*_i_	Local dry hydrogel retention density in core segment i
*k*	Core permeability
*Q*	Volumetric flow rate
*μ*	Fluid viscosity
*L*	Core length
*A*	Core cross-sectional area
Δ*P*	Pressure drop
*F*_j_	Flow fraction of branch j
*Q*_H_	Effluent flow rate from the high-permeability branch
*Q*_L_	Effluent flow rate from the low-permeability branch
