# Supplementary material for: Pore-Throat-Adaptive Transport and Flow-Redistribution Mechanisms of Deformable Hydrogel Particles in Tight Reservoirs of the Ordos Basin
Source: Gels. 2026 Oct 14;12(10):924. doi: 10.3390/gels12100924 (PMC13704001; doi:10.3390/gels12100924)
Supplement: Supplementary file 1 [file gels-12-00924-s001.zip › gels-4579841-supplementary.pdf]

Table S1. Physical and mineralogical properties of individual Ordos tight sandstone cores used in this study

Core ID	Use	Class	D / mm	L / mm	Porosity / %	k / mD	PV / mL	Quartz / wt%	Feldspar / wt%	Clay / wt%	Carbonates / wt%
CHAR-L-1	Characterization	OTS-L	25.02	49.81	11.13	0.366	2.73	63.63	18.25	13.25	4.88
CHAR-L-2	Characterization	OTS-L	24.93	49.97	11.64	0.412	2.84	63.21	17.34	12.28	7.17
CHAR-L-3	Characterization	OTS-L	25.02	50.02	12.15	0.458	2.99	63.76	17.31	13.13	5.81
CHAR-M-1	Characterization	OTS-M	25.03	49.94	9.02	0.188	2.22	62.11	17.93	12.64	7.33
CHAR-M-2	Characterization	OTS-M	25.06	50.04	9.46	0.216	2.33	62.47	19.16	13.03	5.34
CHAR-M-3	Characterization	OTS-M	25.01	50.13	9.90	0.244	2.44	62.54	18.79	12.56	6.11
CHAR-H-1	Characterization	OTS-H	24.96	49.92	7.46	0.071	1.82	60.98	17.89	14.29	6.84
CHAR-H-2	Characterization	OTS-H	24.98	49.95	7.82	0.083	1.91	62.49	18.41	14.25	4.85
CHAR-H-3	Characterization	OTS-H	24.95	50.09	8.18	0.095	2.00	62.58	18.40	14.18	4.84
SC-L-1	Single-core DHP-M	OTS-L	25.03	50.14	11.15	0.365	2.75	62.36	17.74	12.37	7.53
SC-L-2	Single-core DHP-M	OTS-L	24.95	50.18	11.69	0.418	2.87	63.48	17.85	13.28	5.39
SC-L-3	Single-core DHP-M	OTS-L	24.99	49.91	12.11	0.451	2.96	62.77	18.12	13.26	5.85
SC-M-1	Single-core DHP-M	OTS-M	24.98	49.89	9.05	0.188	2.21	61.66	17.87	12.65	7.82
SC-M-2	Single-core DHP-M	OTS-M	25.02	50.12	9.48	0.218	2.34	62.19	19.19	13.24	5.38
SC-M-3	Single-core DHP-M	OTS-M	25.08	50.14	9.85	0.241	2.44	62.73	18.55	12.87	5.85
SC-H-1	Single-core DHP-M	OTS-H	25.02	49.84	7.46	0.071	1.83	62.52	18.73	13.77	4.99
SC-H-2	Single-core DHP-M	OTS-H	25.00	49.87	7.86	0.086	1.92	61.34	18.39	14.25	6.02
SC-H-3	Single-core DHP-M	OTS-H	24.94	50.11	8.16	0.094	2.00	61.75	17.50	13.85	6.89
SIZE-S-1	Size comparison DHP-S	OTS-M	24.92	50.17	9.04	0.189	2.21	62.05	17.88	13.73	6.34

SIZE-S-2	Size comparison DHP-S	OTS-M	25.07	49.83	9.48	0.218	2.33	62.87	18.87	12.68	5.58
SIZE-S-3	Size comparison DHP-S	OTS-M	25.00	50.02	9.86	0.241	2.42	62.26	18.10	13.26	6.39
SIZE-M-1	Size comparison DHP-M	OTS-M	25.04	49.88	9.01	0.190	2.21	62.67	18.41	13.22	5.69
SIZE-M-2	Size comparison DHP-M	OTS-M	24.94	49.89	9.47	0.221	2.31	61.91	18.11	12.60	7.38
SIZE-M-3	Size comparison DHP-M	OTS-M	25.02	49.89	9.90	0.241	2.43	61.63	18.13	13.44	6.80
SIZE-L-1	Size comparison DHP-L	OTS-M	24.95	49.85	9.05	0.188	2.21	62.35	18.90	13.63	5.12
SIZE-L-2	Size comparison DHP-L	OTS-M	24.95	49.84	9.48	0.220	2.31	62.34	18.82	13.44	5.40
SIZE-L-3	Size comparison DHP-L	OTS-M	25.08	49.84	9.87	0.239	2.43	63.05	18.15	12.77	6.03
FD-LM-HF1	FD-LM high-flow	OTS-L	24.99	49.97	11.12	0.365	2.72	63.96	17.92	13.21	4.91
FD-LM-HF2	FD-LM high-flow	OTS-L	25.01	49.82	11.70	0.420	2.86	64.04	18.60	13.19	4.17
FD-LM-HF3	FD-LM high-flow	OTS-L	24.95	49.99	12.11	0.451	2.96	62.41	17.83	13.38	6.38
FD-LM-LF1	FD-LM low-flow	OTS-M	24.96	50.11	9.02	0.188	2.21	63.22	19.19	13.28	4.31
FD-LM-LF2	FD-LM low-flow	OTS-M	25.03	49.86	9.47	0.222	2.32	62.54	18.56	13.55	5.35
FD-LM-LF3	FD-LM low-flow	OTS-M	24.93	50.03	9.88	0.241	2.41	61.78	19.15	12.61	6.46
FD-LH-HF1	FD-LH high-flow	OTS-L	24.95	50.04	11.14	0.365	2.73	62.52	18.55	12.34	6.59
FD-LH-HF2	FD-LH high-flow	OTS-L	25.02	50.05	11.66	0.419	2.87	63.24	18.61	12.29	5.86
FD-LH-HF3	FD-LH high-flow	OTS-L	25.03	49.90	12.12	0.452	2.98	62.84	17.74	13.05	6.36

FD-LH-LF1	FD-LH low-flow	OTS-H	24.93	49.98	7.49	0.073	1.83	60.93	17.80	13.77	7.50
FD-LH-LF2	FD-LH low-flow	OTS-H	25.07	50.15	7.86	0.084	1.95	61.08	18.67	14.38	5.86
FD-LH-LF3	FD-LH low-flow	OTS-H	25.02	50.19	8.17	0.091	2.02	62.27	17.92	14.33	5.48
FD-MH-HF1	FD-MH high-flow	OTS-M	25.07	49.85	9.00	0.186	2.21	62.50	18.18	13.35	5.98
FD-MH-HF2	FD-MH high-flow	OTS-M	25.03	49.88	9.49	0.219	2.33	62.38	19.07	13.68	4.87
FD-MH-HF3	FD-MH high-flow	OTS-M	24.93	49.97	9.86	0.238	2.41	62.89	18.69	12.87	5.55
FD-MH-LF1	FD-MH low-flow	OTS-H	25.04	50.02	7.46	0.069	1.84	60.94	18.74	14.67	5.66
FD-MH-LF2	FD-MH low-flow	OTS-H	25.01	50.13	7.84	0.083	1.93	61.03	17.93	14.66	6.38
FD-MH-LF3	FD-MH low-flow	OTS-H	25.05	50.14	8.19	0.092	2.02	61.25	17.64	14.49	6.61
EOR-LM-HF1	EOR-LM high-flow	OTS-L	25.06	49.96	11.14	0.365	2.74	63.97	18.51	13.37	4.15
EOR-LM-HF2	EOR-LM high-flow	OTS-L	25.05	50.15	11.64	0.420	2.88	62.90	18.60	13.12	5.38
EOR-LM-HF3	EOR-LM high-flow	OTS-L	25.06	50.12	12.11	0.452	2.99	62.49	18.52	13.20	5.78
EOR-LM-LF1	EOR-LM low-flow	OTS-M	24.96	50.13	9.02	0.187	2.21	62.93	18.12	12.53	6.42
EOR-LM-LF2	EOR-LM low-flow	OTS-M	24.95	49.93	9.50	0.222	2.32	62.00	18.70	13.06	6.24

EOR-LM-LF3	EOR-LM low-flow	OTS-M	25.08	50.01	9.90	0.238	2.45	63.25	18.05	13.85	4.86
EOR-LH-HF1	EOR-LH high-flow	OTS-L	24.96	49.84	11.13	0.367	2.71	62.86	18.15	12.72	6.27
EOR-LH-HF2	EOR-LH high-flow	OTS-L	24.98	50.03	11.65	0.420	2.86	62.30	18.60	12.75	6.35
EOR-LH-HF3	EOR-LH high-flow	OTS-L	25.04	50.10	12.13	0.451	2.99	62.43	18.23	12.46	6.88
EOR-LH-LF1	EOR-LH low-flow	OTS-H	24.97	50.14	7.47	0.070	1.83	61.36	18.07	13.96	6.61
EOR-LH-LF2	EOR-LH low-flow	OTS-H	24.97	49.85	7.83	0.087	1.91	62.02	18.76	14.26	4.96
EOR-LH-LF3	EOR-LH low-flow	OTS-H	24.97	50.02	8.13	0.092	1.99	61.57	18.31	14.32	5.80
EOR-MH-HF1	EOR-MH high-flow	OTS-M	24.99	49.98	9.00	0.188	2.21	63.12	18.91	12.74	5.23
EOR-MH-HF2	EOR-MH high-flow	OTS-M	24.93	50.01	9.49	0.220	2.32	62.97	18.85	13.44	4.73
EOR-MH-HF3	EOR-MH high-flow	OTS-M	24.96	49.88	9.85	0.239	2.40	62.36	18.99	12.60	6.05
EOR-MH-LF1	EOR-MH low-flow	OTS-H	24.99	50.05	7.44	0.072	1.83	61.69	17.84	14.32	6.15
EOR-MH-LF2	EOR-MH low-flow	OTS-H	24.92	50.10	7.85	0.083	1.92	61.57	17.75	14.74	5.95
EOR-MH-LF3	EOR-MH low-flow	OTS-H	25.00	49.82	8.15	0.095	1.99	61.62	18.62	14.33	5.42
CTRL-LM-HF1	Untreated control, HCS-LM high-flow	OTS-L	25.01	49.90	11.18	0.372	2.74	63.1	18.2	12.9	5.8

CTRL-LM-HF2	Untreated control, HCS-LM high-flow	OTS-L	24.97	50.08	11.66	0.414	2.86	62.8	18.5	13.1	5.6
CTRL-LM-HF3	Untreated control, HCS-LM high-flow	OTS-L	25.04	50.03	12.08	0.449	2.98	63.4	17.9	12.8	5.9
CTRL-LM-LF1	Untreated control, HCS-LM low-flow	OTS-M	24.96	49.92	9.06	0.191	2.22	62.2	18.1	13.0	6.7
CTRL-LM-LF2	Untreated control, HCS-LM low-flow	OTS-M	25.02	50.06	9.44	0.215	2.33	62.6	18.7	13.2	5.5
CTRL-LM-LF3	Untreated control, HCS-LM low-flow	OTS-M	25.05	49.95	9.88	0.242	2.43	62.1	18.5	12.7	6.7
CTRL-LH-HF1	Untreated control, HCS-LH high-flow	OTS-L	24.95	50.10	11.20	0.374	2.74	63.0	18.0	13.0	6.0
CTRL-LH-HF2	Untreated control, HCS-LH high-flow	OTS-L	25.06	49.88	11.63	0.417	2.87	62.7	18.4	12.7	6.2
CTRL-LH-HF3	Untreated control, HCS-LH high-flow	OTS-L	25.00	50.12	12.09	0.447	2.98	63.2	18.1	12.9	5.8
CTRL-LH-LF1	Untreated control, HCS-LH low-flow	OTS-H	24.94	49.93	7.50	0.073	1.83	61.4	18.0	14.1	6.5
CTRL-LH-LF2	Untreated control, HCS-LH low-flow	OTS-H	25.03	50.07	7.81	0.082	1.92	61.8	18.4	14.3	5.5
CTRL-LH-LF3	Untreated control, HCS-LH low-flow	OTS-H	25.07	49.98	8.15	0.094	2.01	61.2	17.9	14.5	6.4
CTRL-MH-HF1	Untreated control, HCS-MH high-flow	OTS-M	24.98	50.04	9.04	0.190	2.22	62.4	18.2	13.1	6.3
CTRL-MH-HF2	Untreated control, HCS-MH high-flow	OTS-M	25.04	49.89	9.45	0.217	2.33	62.0	18.8	12.9	6.3
CTRL-MH-HF3	Untreated control, HCS-MH high-flow	OTS-M	24.93	50.11	9.87	0.240	2.42	62.5	18.4	12.6	6.5

CTRL-MH-LF1	Untreated control, HCS-MH low-flow	OTS-H	25.02	49.96	7.48	0.071	1.84	61.6	18.1	14.0	6.3
CTRL-MH-LF2	Untreated control, HCS-MH low-flow	OTS-H	24.96	50.09	7.84	0.084	1.92	61.3	18.5	14.4	5.8
CTRL-MH-LF3	Untreated control, HCS-MH low-flow	OTS-H	25.05	50.02	8.14	0.093	2.01	61.9	17.8	14.2	6.1

Table S2. Actual hydrogel injection volumes and dry hydrogel masses used in the core-flooding experiments

Run ID	Experiment	Core or assembly PV / mL	Actual DHP injection / PV	Dispersion volume / mL	Dry DHP mass / mg	Early termination
SC-L-1	DHP-M single-core, OTS-L	2.75	0.30	0.825	2.47	No
SC-L-2	DHP-M single-core, OTS-L	2.87	0.30	0.861	2.58	No
SC-L-3	DHP-M single-core, OTS-L	2.96	0.30	0.888	2.66	No
SC-M-1	DHP-M single-core, OTS-M	2.21	0.30	0.663	1.99	No
SC-M-2	DHP-M single-core, OTS-M	2.34	0.30	0.702	2.11	No
SC-M-3	DHP-M single-core, OTS-M	2.44	0.30	0.732	2.20	No
SC-H-1	DHP-M single-core, OTS-H	1.83	0.30	0.549	1.65	No
SC-H-2	DHP-M single-core, OTS-H	1.92	0.30	0.576	1.73	No
SC-H-3	DHP-M single-core, OTS-H	2.00	0.30	0.600	1.80	No
SIZE-S-1	DHP-S in matched OTS-M	2.21	0.30	0.663	1.99	No
SIZE-S-2	DHP-S in matched OTS-M	2.33	0.30	0.699	2.10	No
SIZE-S-3	DHP-S in matched OTS-M	2.42	0.30	0.726	2.18	No
SIZE-M-1	DHP-M in matched OTS-M	2.21	0.30	0.663	1.99	No
SIZE-M-2	DHP-M in matched OTS-M	2.31	0.30	0.693	2.08	No

SIZE-M-3	DHP-M in matched OTS-M	2.43	0.30	0.729	2.19	No
SIZE-L-1	DHP-L in matched OTS-M	2.21	0.30	0.663	1.99	No
SIZE-L-2	DHP-L in matched OTS-M	2.31	0.30	0.693	2.08	No
SIZE-L-3	DHP-L in matched OTS-M	2.43	0.30	0.729	2.19	No
FD-LM-1	HCS-LM flow diversion	4.93	0.30	1.479	4.44	No
FD-LM-2	HCS-LM flow diversion	5.18	0.30	1.554	4.66	No
FD-LM-3	HCS-LM flow diversion	5.37	0.30	1.611	4.83	No
FD-LH-1	HCS-LH flow diversion	4.56	0.30	1.368	4.10	No
FD-LH-2	HCS-LH flow diversion	4.82	0.30	1.446	4.34	No
FD-LH-3	HCS-LH flow diversion	5.00	0.30	1.500	4.50	No
FD-MH-1	HCS-MH flow diversion	4.05	0.30	1.215	3.64	No
FD-MH-2	HCS-MH flow diversion	4.26	0.30	1.278	3.83	No
FD-MH-3	HCS-MH flow diversion	4.43	0.30	1.329	3.99	No
EOR-LM-1	HCS-LM displacement	4.95	0.30	1.485	4.46	No
EOR-LM-2	HCS-LM displacement	5.20	0.30	1.560	4.68	No
EOR-LM-3	HCS-LM displacement	5.44	0.30	1.632	4.90	No
EOR-LH-1	HCS-LH displacement	4.54	0.30	1.362	4.09	No
EOR-LH-2	HCS-LH displacement	4.77	0.30	1.431	4.29	No
EOR-LH-3	HCS-LH displacement	4.98	0.30	1.494	4.48	No
EOR-MH-1	HCS-MH displacement	4.04	0.30	1.212	3.64	No
EOR-MH-2	HCS-MH displacement	4.24	0.30	1.272	3.82	No
EOR-MH-3	HCS-MH displacement	4.39	0.30	1.317	3.95	No

Table S3. Comparison of DHP-M-treated and untreated control experiments in heterogeneous parallel-core systems

System	Treatment	Increase in low-flow movable-fluid saturation / %	Increase in low-flow flow fraction / %	Incremental oil recovery / %
HCS-LM	Control	3.2 ± 0.7	2.9 ± 0.6	2.3 ± 0.4
HCS-LM	DHP-M	17.6 ± 2.1	17.6 ± 2.5	11.8 ± 1.2
HCS-LH	Control	2.5 ± 0.6	2.2 ± 0.5	1.7 ± 0.3
HCS-LH	DHP-M	24.9 ± 2.4	24.9 ± 2.9	16.4 ± 1.5
HCS-MH	Control	2.8 ± 0.6	2.5 ± 0.5	2.0 ± 0.4
HCS-MH	DHP-M	19.9 ± 2.2	19.9 ± 2.7	15.8 ± 1.4

Table S4. Quantitative micro-CT parameters of representative Ordos tight sandstone cores before and after DHP-M treatment.

Core group	Connected porosity before / %	Connected porosity after / %	Relative change / %	Tortuosity before	Tortuosity after	Relative change / %
OTS-L	7.26	6.95	-4.3	1.41	1.47	+4.3
OTS-M	4.42	3.84	-13.1	1.69	1.95	+15.4
OTS-H	1.86	1.74	-6.5	2.12	2.23	+5.2

Table S5. Effect of injection rate on DHP-M transport in matched OTS-M cores.

Injection rate / (mL/min)	Peak pressure drop / MPa	Peak apparent pressure gradient / MPa·m^{-1}	Particle-retention efficiency / %	Cumulative effluent particle recovery / %
0.10	0.46 ± 0.01	9.2 ± 0.2	85.4 ± 1.5	16.1 ± 1.4
0.20	0.69 ± 0.03	13.8 ± 0.6	78.0 ± 2.6	24.0 ± 2.0
0.30	0.91 ± 0.04	18.2 ± 0.7	69.0 ± 2.1	32.6 ± 2.6

Table S6. Rheological properties of 0.30 wt% DHP-M dispersions at 60 °C under different salinity conditions.

Salinity / (mg/L)	Apparent viscosity at 10 s^{-1} / mPa·s	Apparent viscosity at 100 s^{-1} / mPa·s	Flow behavior index *n*
50,000	3.84 ± 0.18	2.71 ± 0.14	0.86 ± 0.02
100,000	3.46 ± 0.16	2.52 ± 0.12	0.88 ± 0.02
150,000	3.12 ± 0.15	2.34 ± 0.11	0.90 ± 0.02

Table S7. Sensitivity of the apparent confinement coefficient λapp to the prescribed bulk-strain reference level

Hydrogel–core combination	0%	20%	30%	40%	50%
DHP-S / OTS-L	47.0	37.6	32.9	28.2	23.5
DHP-S / OTS-M	85.7	68.6	60.0	51.4	42.9
DHP-S / OTS-H	161.9	129.5	113.3	97.1	81.0
DHP-M / OTS-L	95.6	76.5	66.9	57.4	47.8
DHP-M / OTS-M	174.4	139.5	122.1	104.6	87.2
DHP-M / OTS-H	329.4	263.5	230.6	197.6	164.7
DHP-L / OTS-L	181.2	145.0	126.8	108.7	90.6
DHP-L / OTS-M	330.4	264.3	231.3	198.2	165.2
DHP-L / OTS-H	624.2	499.4	436.9	374.5	312.1
